# Netrin-1 promotes glioma growth by activating NF-κB via UNC5A

**DOI:** 10.1038/s41598-017-05707-0

**Published:** 2017-07-14

**Authors:** Jing-Ying Chen, Xiao-Xiao He, Chi Ma, Xin-Min Wu, Xi-Lin Wan, Zhen-Kai Xing, Qing-Qing Pei, Xian-Ping Dong, Dong-Xu Liu, Wen-Cheng Xiong, Xiao-Juan Zhu

**Affiliations:** 1 Key Laboratory of Molecular Epigenetics of Ministry of Education, Institute of Cytology and Genetics, Northeast Normal University, Changchun, 130024 China; 2 Department of Neurosurgery, First Hospital of Jilin University, Changchun, China; 3 Department of Physiology and Biophysics, Dalhousie University, Halifax, Canada; 4 School of Science, Faculty of Health and Environmental Sciences, Auckland University of Technology, Auckland, 1010 New Zealand; 5 Department of Neuroscience & Regenerative Medicine and Department of Neurology, Medical College of Georgia, Augusta University, Augusta, Georgia 30912, USA

## Abstract

Gliomas, a common type of brain tumor, are characterized by aggressive infiltration, making it difficultly to cure by surgery. Netrin-1, an extracellular guidance cue critical for neuronal axon path-finding, has been reported to play an important role in cell invasion and migration in several types of cancers. However, the role of netrin-1 in glioma remains largely unknown. Here, we provide evidence suggested that Netrin-1 has a critical role in glioma growth. We found that netrin-1 was significantly increased in glioma samples and positively correlated with cell proliferation, tumor grade and malignancy. Netrin-1 knockdown reduced cell proliferation and attenuated tumor growth in a xenograft mouse model. Further studies found that netrin-1 induced NF-κB p65^ser536^ phosphorylation and c-Myc expression *in vitro* and *in vivo*. Interestingly, activation of NF-κB by netrin-1 was dependent on UNC5A receptor, because suppression of UNC5A significantly inhibited NF-κB p65^ser536^ phosphorylation, c-Myc up-regulation and reduced cell proliferation. Taken together, these results suggested netrin-1 promotes glioma cell proliferation by activating NF-κB signaling via UNC5A, netrin-1 may be a potential therapeutic target for the treatment of glioma.

## Introduction

Gliomas are the most prevalent primary brain tumors^1^ and characterized by prominent proliferation and aggressive infiltration. Therefore, gliomas often extend into normal brain tissues with no clear margins^2, 3^. Gliomas can be classified into four grades: I, II, III, and IV, according to the World Health Organization classification for tumors of the central nervous system^4^. The median prognosis for the most malignant gliomas (grade IV: glioblastoma) is less than 15–20 months^3^. In the last two decades, a variety of genetic abnormalities and aberrant cell signaling pathways have been implicated in glioma tumorigenesis, including amplification of *VEGF* ^5^, loss of *PTEN* ^1, 6–8^, dysfunction of *p53* ^1, 7^, and mutations of *NF1* ^1, 7^.

Netrin-1 is a member of the laminin-related family of matrix-binding secreted proteins. It was first described as a guidance molecule for neuronal axon path-finding. Netrin-1 function was mediated by its receptors, such as DCC and UNC5 homologue family members^9–11^. These canonical receptors promote cell death when netrin-1 is absent but enhance cell survival when netrin-1 is present^9, 10, 12^. Recently, netrin-1 was proposed to regulate cell survival and tumorigenesis^9, 13–16^. Accumulating evidence suggested that netrin-1 is a potential oncogene as it has been shown to be up-regulated in many types of cancer^17–25^, such as pediatric medulloblastoma^18^, non-small cell lung cancer^19^, primary breast tumors^22^, hepatocyte carcinoma^24^, bladder cancer^25^, and glioblastoma^26, 27^, as well as in a large fraction of aggressive neuroblastomas^20^. Shimizu and colleagues have shown that netrin-1 could activate RhoA and cathepsin B to promote glioblastoma cell migration and angiogenesis^26^. Overexpression of netrin-1 could activate Notch signaling to mediate glioblastoma cell invasion^27^. In addition, combining chemotherapeutic agents and netrin-1 interference potentiates cancer cell death^28^. Although netrin-1 has been implicated in cancer cell survival and cell motility, there is little research regarding its role in cancer cell proliferation. Given that netrin-1 plays a role in maintaining cell proliferation in the ventricular zone of the developing brainstem^29^, it is interesting to test whether netrin-1 promotes glioma cell proliferation.

In the present study, we showed that netrin-1 was up-regulated in glioma tissues. Netrin-1 expression was positively associated with the cell proliferation marker Ki-67, tumor grade, and tumor malignancy. The addition of netrin-1 effectively enhanced cell proliferation, whereas knockdown of netrin-1 expression greatly reduced tumorigenesis *in vitro* and *in vivo*. We also demonstrated that netrin-1 activated c-Myc expression through the NF-κB pathway via the UNC5A receptor. These results suggest that netrin-1 is an important factor for glioma cell proliferation, and it may be a promising target for glioma treatment.

## Results

### Increased netrin-1 expression in glioma tissue specimens

**Figure 1:**
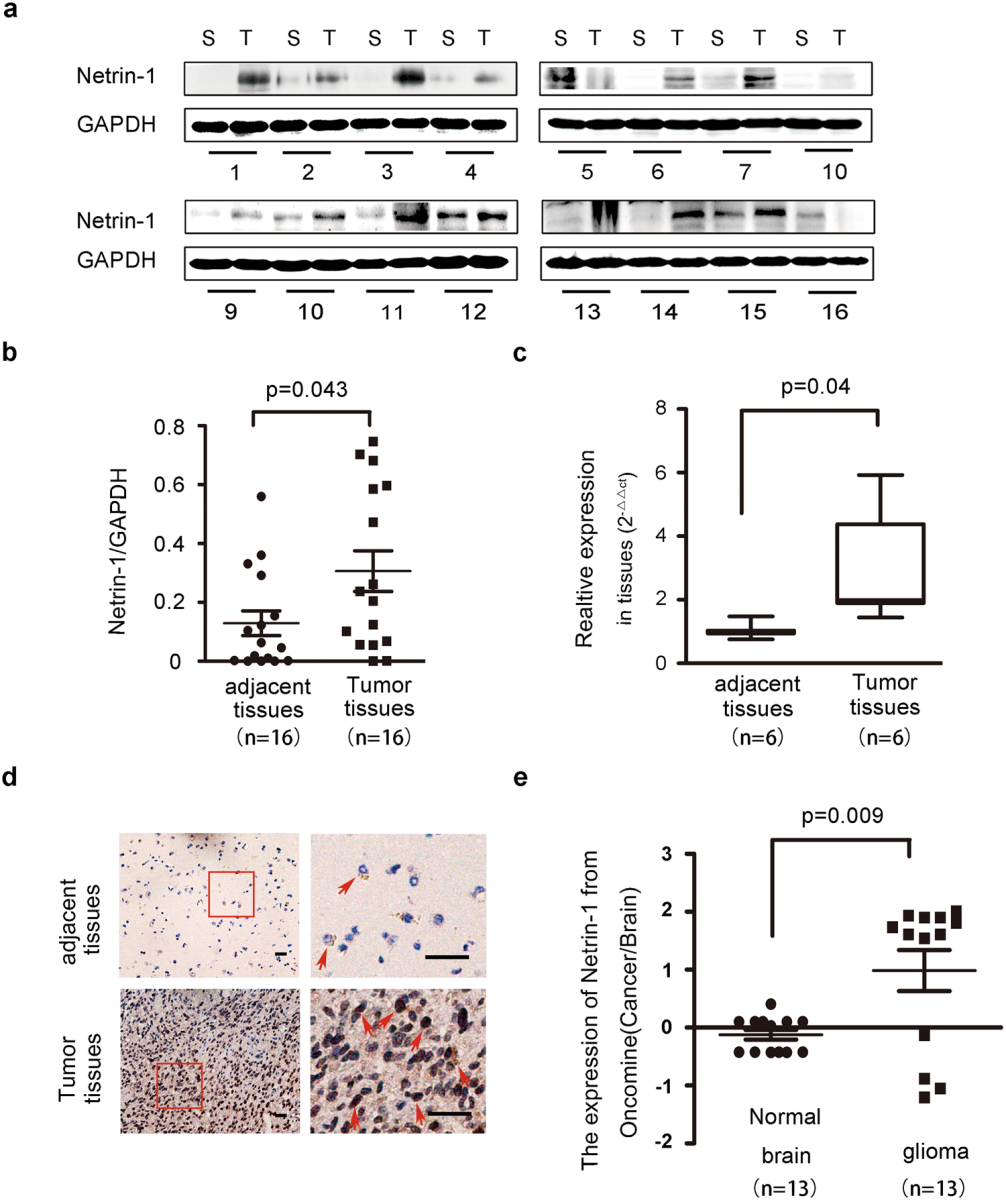
Netrin-1 expression was elevated in glioma tissue specimens. (**a**) Western blotting analyses of netrin-1 expression in fresh tumor tissues (T) and their adjacent normal tissues (S) from 16 patients (No. 1–16). Netrin-1 expression was significantly higher in glioma tumor tissues. GAPDH was used as a loading control. (**b**) Quantification of netrin-1 expression using Gel-Pro Analyzer 4 software. The values indicated the relative netrin-1 expression in the fresh samples. (**c**) Real-time PCR analysis of *netrin-1* expression in 6 paired glioma tissues and adjacent normal brain tissues. The results represent data from three independent experiments. (**d**) IHC staining of human glioma cancer samples showing netrin-1 expression. Boxed areas in the left row are magnified and shown in the right row. Red arrowheads indicated examples of cells with positive netrin-1 staining. Scale bar: 50 μm. (**e**) Expression analysis of netrin-1 in normal brain and glioma samples from the Oncomine cDNA microarray database. The data are shown as the mean ± SEM.

To assess the importance of netrin-1 in glioma tumorigenesis, we firstly measured netrin-1 expression in 16 paired fresh glioma specimens. Western blotting analysis revealed that netrin-1 expression was higher in 87.5% (14/16) of tumor tissues compared with their paired adjacent normal tissues (n = 16, p = 0.043; Fig. 1a,b, Supplementary Table S1). Real-time PCR assay also revealed an average of 2.966-fold (n = 6, p = 0.04; Fig. 1c) increase of *netrin-1* mRNA in glioma tissues. Moreover, approximately 70% of the tumor cells stained positively for the netrin-1 protein in immunohistochemistry (IHC) (Fig. 1d), whereas positive staining of netrin-1 in normal brain cells was hardly observed. These data indicated that netrin-1 expression was significantly up-regulated in the glioma.

We further analyzed *netrin-1* expression in the Oncomine cDNA microarray database. Oncomine currently contains 13 glioma microarray datasets. Elevated *netrin-1* expression in gliomas, ranging from 2.16- to 4.545-fold (Supplementary Table S2), was reported in 7 of the datasets including anaplastic astrocytoma (1 dataset, n = 42), anaplastic oligoastrocytoma (2 datasets, n = 39), oligodendroglioma (1 dataset, n = 73), diffuse astrocytoma (1 dataset, n = 30) and glioblastoma (2 datasets, n = 188). Although decreased *netrin-1* expression was reported in six other datasets, the fold change was less than 2, ranging from 1.119- to 1.807-fold. The Oncomine data supported our findings that *netrin-1* expression was elevated in clinical glioma samples (Fig. 1e and Supplementary Table S2).

### Positive correlation of netrin-1 expression with cell proliferation in glioma tissue specimens

**Figure 2:**
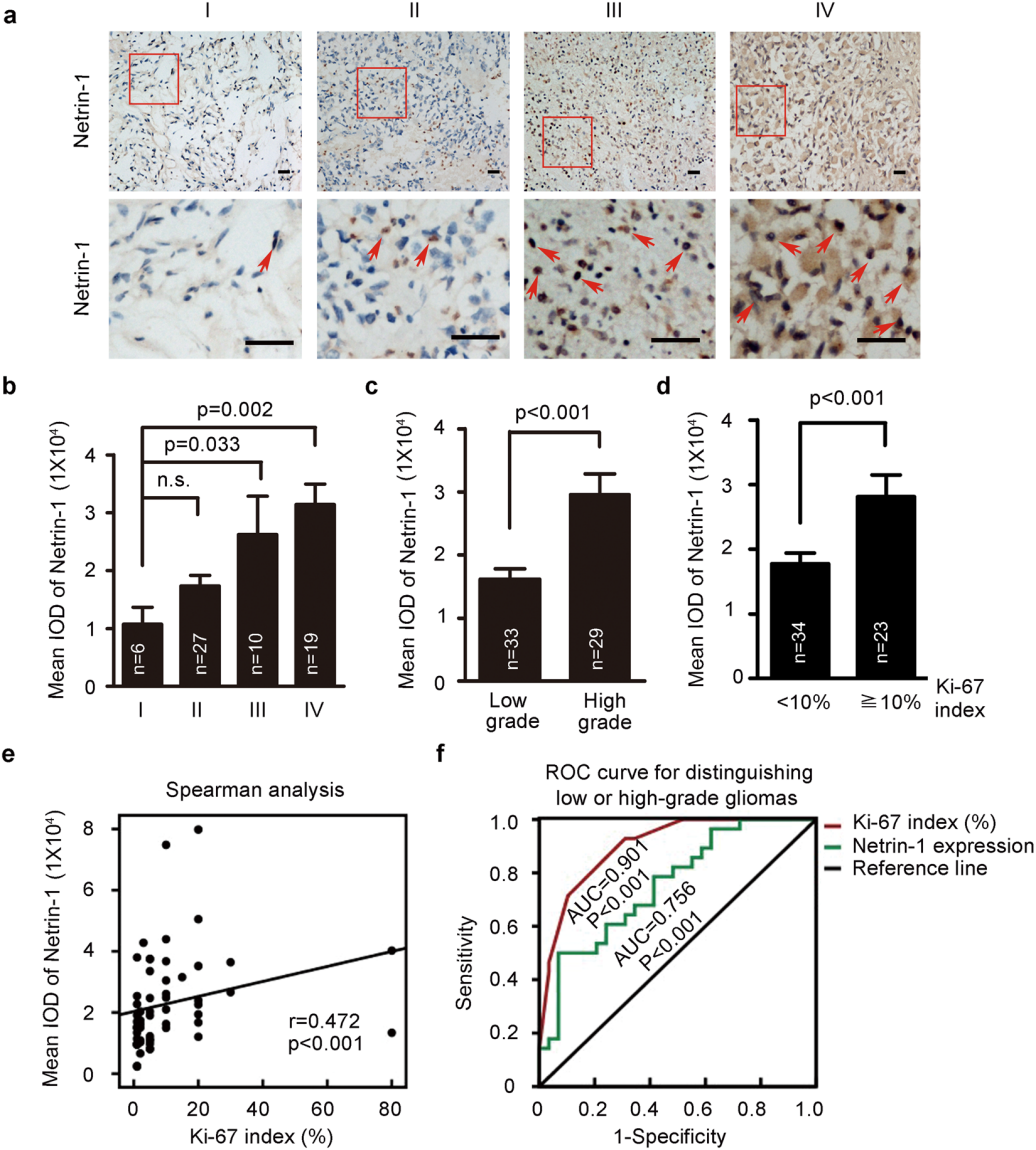
Elevated netrin-1 expression was positively correlated with cell proliferation in glioma tissue specimens. (**a**) Representative images of netrin-1 staining by IHC in glioma cancer sections. Boxed areas in the upper panels were magnified and are shown in the corresponding lower panels. Red arrowheads indicated examples of cells with positive netrin-1 staining. Scale bar: 50 μm. (**b**) Relative netrin-1 expression in glioma specimens of different grades. The mean integrated optical density (IOD) of netrin-1 staining was determined using Image-Pro Plus. (**c**) Relative netrin-1 expression in low- and high-grade gliomas. The mean IOD of netrin-1 staining was determined using Image-Pro Plus. (**d**) Ki-67 expression in low- and high-grade gliomas. (**e**) Netrin-1 expression and Ki-67 index of each glioma tumor sample were plotted for the Spearman analysis. (**f**) ROC analysis showed that netrin-1 expression (AUC = 0.756, p < 0.001) and Ki-67 levels (AUC = 0.901, p < 0.001) could discriminate low-grade gliomas from high-grade gliomas. One-way ANOVA and Student’s t-test were used to test for statistical significance. The data are shown as the mean ± SEM. n.s., P > 0.05.

To study the correlation between netrin-1 and glioma progression, 62 formalin-fixed and paraffin-embedded glioma specimens, including grade I, II, III and IV, were analyzed for netrin-1 staining by IHC (see Supplementary Table S1 for sample details). These glioma specimens were obtained from clinical patients who did not receive chemotherapy before surgery. Two senior pathologists evaluated each slide for determining histology grades (I, II, III and IV) and glioma parameters, such as Ki-67 index, MGMT expression and tumor latency. The average staining intensity of netrin-1 showed a steady increasing trend from grade I to grade IV (Fig. 2a,b). Furthermore, using the median IHC staining reading as a threshold, individual samples that had higher IHC staining readings were mostly grade III and IV gliomas, with few grade II samples and hardly any grade I samples (Supplementary Fig. S1). To validate the association between netrin-1 and glioma recurrence, we assessed netrin-1 expression in paired primary and recurrent samples (n = 16, Supplementary Table S3) using IHC. Netrin-1 expression was substantially higher in recurrent glioma than primary glioma specimens (Supplementary Fig. S2a). Clinically, grade I and grade II gliomas are classified as low-grade gliomas, while grade III and grade IV gliomas are classified as high-grade gliomas. The higher netrin-1 staining observed in high-grade gliomas and recurrent glioma further suggested that netrin-1 was an important factor in glioma progression (Fig. 2c and Supplementary Fig. S2b).

After identifying a correlation between netrin-1 and glioma malignancy, we next investigated the correlation of netrin-1 expression with several known glioma parameters. According to the Melling and von Wasielewski’s category method, 0–10% Ki-67 positive staining is considered low Ki-67, whereas 10% and higher Ki-67 positive staining is considered high Ki-67^30, 31^. Among these samples, 54.8% of patients (34/62) had a low Ki-67 index, 37.1% of patients (23/62) had a high Ki-67 index, and 8.1% of patients (5/62) had no available data. Similarly, samples with a high Ki-67 index mostly had been categorized as grade III and IV gliomas. Our data exhibited a positive correlation between netrin-1 expression and Ki-67 index (Fig. 2d). A moderate positive correlation between netrin-1 expression and Ki-67 index was found determined by Spearman correlation coefficient (Fig. 2e, r = 0.472, P < 0.001). No significant correlations were found for gender (p = 0.166), age (p = 0.881), or MGMT levels (p = 0.819; Table 1).

**Table 1:** The 62 glioma patients’ classified statistic according to clinical pathological features.

Factor	Variable	N (%)	Mean IOD of netrin-1 (Mean ± SEM)	p value*
Histology	Low grade	33 (53.2%)	16124.4309 ± 1645.7586	0.0001^a^
	High grade	29 (46.8%)	29621.221 ± 3218.8488	
Ki-67	<10%	34 (54.8%)	17153.18 ± 1780.11923	0.001^a^
	≥10%	23 (37.1%)	31713.15 ± 3833.27060	
	no data	5 (8.1%)	20022.03 ± 5990.74457	
Grade	I	6 (9.7%)	10757.63 ± 2940.58031	0.002^b^
	II	27 (43.5%)	17317.0533 ± 1844.97835	
	III	10 (16.1%)	26216.543 ± 6649.36802	
	IV	19 (30.7%)	31413.1568 ± 3528.05192	
Gender	Female	28 (45.2%)	25397.0711 ± 3513.94286	0.166^a^
	Male	34 (54.8%)	20000.1071 ± 1958.32778	
Age	<30	20 (32.3%)	20962.1115 ± 3640.92443	0.881^b^
	30–60	36 (58%)	22777.7208 ± 2589.15001	
	>60	3 (4.8%)	25313.575 ± 4097.56715	
MGMT	−	6 (9.7%)	20737.1467 ± 3889.47198	0.819^a^
	+	56 (90.3%)	22261.9496 ± 2172.64999	

^a^Student’s t-test; ^b^One-way ANOVA.

To investigate the possibility that netrin-1 could serve as a potential glioma biomarker, receiver operating characteristics (ROC) curves were constructed and the area under the ROC curve (AUC) was calculated to assess the diagnostic values of netrin-1 expression and Ki-67 index for glioma^32^. Both Ki-67 index and elevated netrin-1 expression were found to be independent factors for distinguishing low-grade gliomas and high-grade gliomas (Fig. 2f). We found that elevated netrin-1 expression discriminated low-grade gliomas from high-grade gliomas (AUC = 0.758, p = 0.025) and distinguished the primary gliomas from recurrent gliomas (AUC = 0.783, p = 0.006) (Supplementary Fig. S2c). Taken together, these clinical data clearly suggested that netrin-1 may act as a diagnostic marker in glioma and play a role in the gliomas proliferation.

### Requirement of netrin-1 expression for cell proliferation in cultured glioma cells

**Figure 3:**
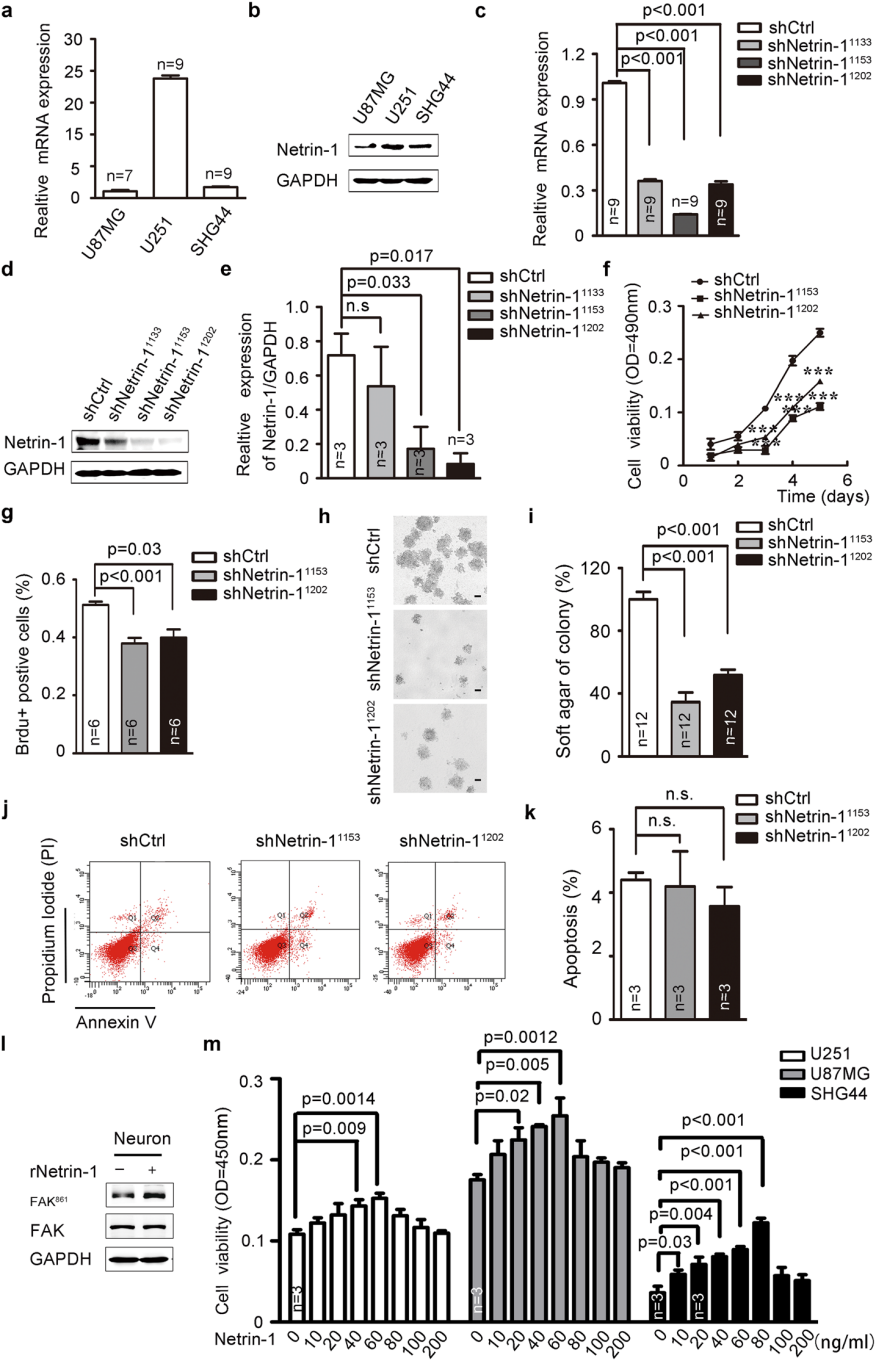
Netrin-1 was required for cell proliferation in cultured glioma cells. (**a** and **b**) Netrin-1 mRNA and protein levels were determined by real-time PCR and western blotting in three cultured glioma cells. (**c** and **d**) Detection of netrin-1 expression by real-time PCR and western blotting in U251 cells infected with lentivirus expressing shCtrl or shNetrin-1. GAPDH was used as a loading control in both assays. (**e**) Quantification of netrin-1 expression using Gel-Pro Analyzer 4 software. (**f**) MTT assays were used to determine the cell viability of U251 cells infected with lentivirus expressing shCtrl or shNetrin-1. (**g**) BrdU analysis was performed to detect cell proliferation in U251 cells infected with lentivirus expressing shCtrl or shNetrin-1. (**h**) U251-shCtrl or shNetrin-1 cells were plated in 0.3% soft agar. Three thousand cells were incubated for 16 days, and the media was changed every 2 days. Cells were counted using a dissecting microscope. Scale bar: 50 μm. (**i**) The colony formation assays were quantified by counting the number of colonies. (**j** and **k**) Apoptosis was measured by flow cytometry of U251 cells after infection with lentivirus expressing shCtrl or shNetrin-1. (**l**) Cells were treated with recombinant netrin-1. Phosphorylated FAK^861^ was detected by western blotting. (**m**) U251, SHG44 and U87MG cells were treated with increasing concentrations of recombinant human netrin-1. Cell viability was determined by CCK-8 assay after the addition of netrin-1 for 48 h. The data are shown as the mean ± SEM. n.s., P > 0.05.

To further explore how netrin-1 promotes glioma progression, we examined the role of netrin-1 in three cultured human glioma cell lines, U251, U87MG and SHG44. Real-time PCR and western blotting analyses showed that netrin-1 expression was detectable in these cells, with higher expression observed in U251 cells and moderate expression in U87MG and SHG44 cells (Fig. 3a,b). These results are consistent with previous studies where U251 cells were found to be more aggressive than U87MG and SHG44 cells^33^. Next, we constructed three lentivirus-delivered shRNAs against *netrin-1* (Netrin-1 shRNA^1133^, netrin-1 shRNA^1153^ and Netrin-1 shRNA^1202^) to knockdown endogenous netrin-1 expression and a scramble shRNA as a control (shCtrl). Netrin-1 expression was reduced by 70% by shRNA delivered via lentivirus infection (Fig. 3c,d,e). The growth of U251 cells was inhibited in a time-dependent manner, when netrin-1 was silenced (Fig. 3f). To validate the effect of netrin-1 on cell proliferation, we employed a BrdU incorporation assay. Compared to shCtrl cells, cells expressing shNetrin-1 had a significantly lower BrdU incorporation rate (Fig. 3g and Supplementary Fig. S3c). Furthermore, netrin-1 knockdown cells had significantly lower number of colonies in soft agar anchorage-independent growth (Fig. 3h,i). However, we did not detect a significant change in apoptosis of netrin-1 knockdown cells (Fig. 3j,k), suggesting that the reduction in cell proliferation we detected was not the result of increased apoptosis. In agreement with previous studies^26, 27^, netrin-1 knockdown also inhibited cell migration and invasion in our experiments (Supplementary Fig. S3a,b).

We incubated recombinant netrin-1 with the glioma cells to examine the effects of netrin-1 on cell proliferation. Netrin-1 was shown to effectively induce FAK^861^ phosphorylation^34^, validating the biological activity of netrin-1 (Fig. 3l). Exogenous netrin-1 enhanced glioma cell proliferation in a dose-dependent manner, and the most effective concentrations were 40–60 ng/ml for U251 cells, 20–60 ng/ml for U87MG cells, and 10–80 ng/ml for SHG44 cells. Netrin-1 inhibited glioma cells proliferation at concentrations above the optimal^35^ (Fig. 3m). Collectively, these results showed that netrin-1 was involved in glioma cell proliferation *in vitro*.

### Necessity of netrin-1 expression for glioma proliferation *in vivo*

**Figure 4:**
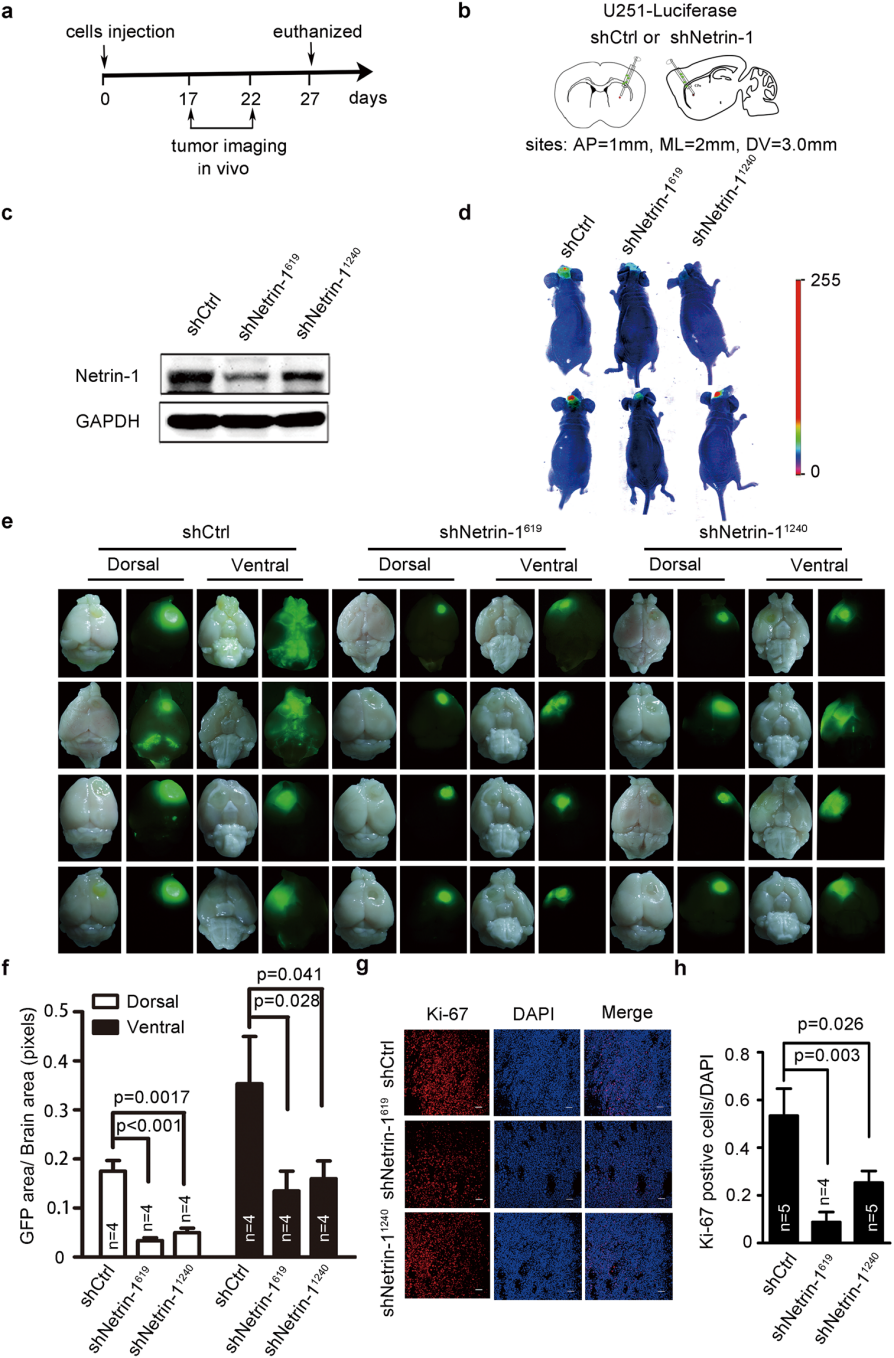
Netrin-1 silencing inhibited glioma growth *in vivo*. (**a**) A schematic diagram of netrin-1 administration. (**b**) A schematic illustration of the injection sites. The red points indicate the injected sites. AP: anterior-posterior of the bregma; ML: medial-lateral of the bregma; DV: dorsal-ventral of the dura. (**c**) Western blotting analyses of netrin-1 expression in U251 cells stably expressing luciferase, Netrin-1 shRNA or control shRNA. (**d**) Images of luciferase signal in tumor-bearing mice visualized by administration of D-Luciferin (15 mg /mL, 200 µl/20 g) potassium salt via intraperitoneal injection. (**e**) Comparison of brain tumor growth in male BALB/c nude mice at the 27^th^ day after implantation of U251 cells infected with lentivirus encoding luciferase together with Netrin-1 shRNA or control shRNA. Dorsal and ventral views of the brain tumors initiated by U251 *in vivo* (n = 4 for each group). (**f**) Quantification of the area of EGFP across the dorsal and ventral brain regions of mice after implantation using Image J software (n = 4 for each group). (**g**) Representative images of Ki-67 staining in U251-derived tumor sections. Scale bar: 50 µm. (**h**) Quantification of Ki-67-positive cells in the three groups (n = 4 for each group). Error bars represent the mean ± SEM.

We next tested if netrin-1 was required for glioma growth. A procedure was set up as shown in Fig. 4a to track the growth of tumor cells *in vivo*. We generated U251 cells that stably expressed the luciferase protein together with EGFP-shNetrin-1 shRNAs (Netrin-1-shRNA^619^ and Netrin-1-shRNA^1240^) or the scramble control EGFP-shRNA (shCtrl). This addressed the issue of weak luciferase expression from the pMT33 viral vector, which was used to monitor the expression of shRNAs in the *in vitro* experiments. Consistent with the results of our earlier shRNAs, these two Netrin-1 shRNAs significantly reduced endogenous netrin-1 expression (Fig. 4c) and inhibited cell proliferation *in vitro* (Supplementary Fig. S4a,b). Next, 4 × 10^5^ cells were stereotactically injected into the right striatum of recipient BALB/c nude mice (n = 12). The projected stereotaxic coordinates were AP (anterior-posterior of the bregma) = 1.0 mm, ML (medial-lateral of the bregma) = 2.0 mm, and DV (medial-lateral of the dura) = 3.0 mm (Fig. 4b). During the following days, *in vivo* tumor formation was monitored by bioluminescence imaging via intraperitoneal injection of luciferin substrate (D-luciferin potassium salt) (Fig. 4a). On the 22^nd^ day after tumor implantation, glioma formation in shCtrl-injected mice was visible, whereas mice injected with either shNetrin-1 exhibited weaker luciferase signals (Fig. 4d). On the 27^th^ day, the mice showed some symptoms characteristic of brain tumors, such as left leg paralysis, lethargy and hypopsia. Therefore, all mice were sacrificed, and the brains were isolated for whole-mount EGFP imaging to visualize tumor size. As shown in Fig. 4e and f, mice injected with shCtrl cells formed clear tumor foci in the right forehead. The tumor foci in the two shCtrl-injected mice even infiltrated into the ventral brain tissue and cerebellum (top left panels, Fig. 4e). In contrast, mice injected with shNetrin-1 cells exhibited relatively smaller tumor foci in the forehead, and no tumor formation was observed in the ventral brain or any other part of the brain (middle and right panels, Fig. 4e).

To evaluate the effect of netrin-1 silencing on cell proliferation *in vivo*, tumor sections were stained for Ki-67 expression (as a marker for proliferating cells) by IHC. We found that the number of Ki-67-positive cells was significantly lower in the shNetrin-1 tumors compared with the shCtrl tumors (Fig. 4g,h). Additionally, TUNEL staining indicated there was no significant increase in apoptosis in tumor tissues *in vivo* (Supplementary Fig. S5a,b). Taken together, these results suggested that netrin-1 also regulated glioma cell proliferation and was involved in glioma tumorigenesis *in vivo*.

### Netrin-1 up-regulation of c-Myc expression in glioma cells

**Figure 5:**
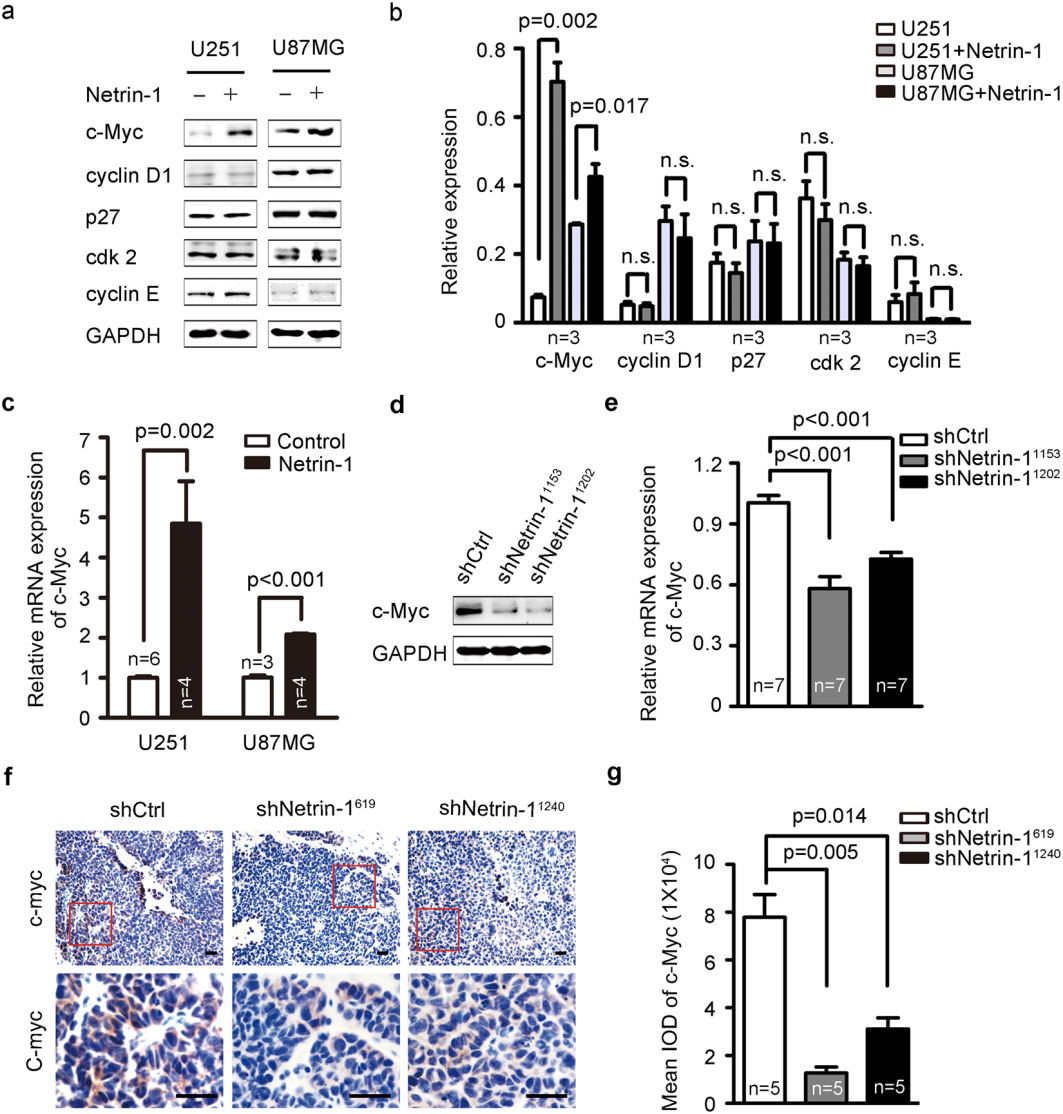
Netrin-1 promoted the expression of the oncogene c-Myc. (**a**) Immunoblotting analyses of the expression of selected oncogenes in U251 and U87MG cells after stimulation with recombinant human netrin-1. (**b**) Quantification of c-Myc, cyclin D1, p27, cdk2 and cyclin E expression using Gel-Pro Analyzer 4 software. (**c**) *c-Myc* expression was detected by real-time PCR in U251 and U87MG cells in the absence or presence of netrin-1. (**d**) Western blotting analyses of c-Myc in U251 cells that were infected with lentivirus expressing shCtrl or shNetrin-1. GAPDH was used as the loading control. (**e**) *c-Myc* expression was detected by real-time PCR in U251 cells that were infected by lentivirus expressing shCtrl or shNetrin-1. (**f**) Representative images of c-Myc staining in U251-derived tumor sections. Boxed areas in the top row defined regions shown at a higher magnification in the bottom row. Scale bar: 50 µm. (**g**) Relative expression of c-Myc in U251-derived tumors. The mean IOD of c-Myc was calculated using Image-Pro Plus.

To identify the molecular mechanisms by which netrin-1 promoted cell proliferation and glioma growth, we examined the expression levels of several key molecules related to cell proliferation and the cell cycle, including *c-Myc*, *cyclin D1, p27*, *cyclin E* and *cdk2* in U251 and U87MG cells. Of the molecules tested, *c-Myc* was significantly up-regulated in response to netrin-1 stimulation. There were no obvious expression changes of cell cycle-related molecules (Fig. 5a,b). This was consistent with our observation that the cell cycle was not altered in netrin-1 knockdown cells (data not shown). Real-time PCR also confirmed that addition of netrin-1 increased *c-Myc* expression (Fig. 5c), and knockdown of netrin-1 decreased c-Myc expression (Fig. 5d,e). In line with our *in vitro* data, we also detected down-regulation of c-Myc in our xenograft shNetrin-1 tumor compared with the shCtrl tumor (Fig. 5f,g). It is well known that c-Myc acts as a signal transducer that promotes cell proliferation in response to signals from the extracellular environment^36^. Therefore, our data suggested that netrin-1 promoted cell proliferation by enhancing c-Myc expression both *in vitro* and *in vivo*.

### Netrin-1 activated NF-κB to induce c-Myc expression

**Figure 6:**
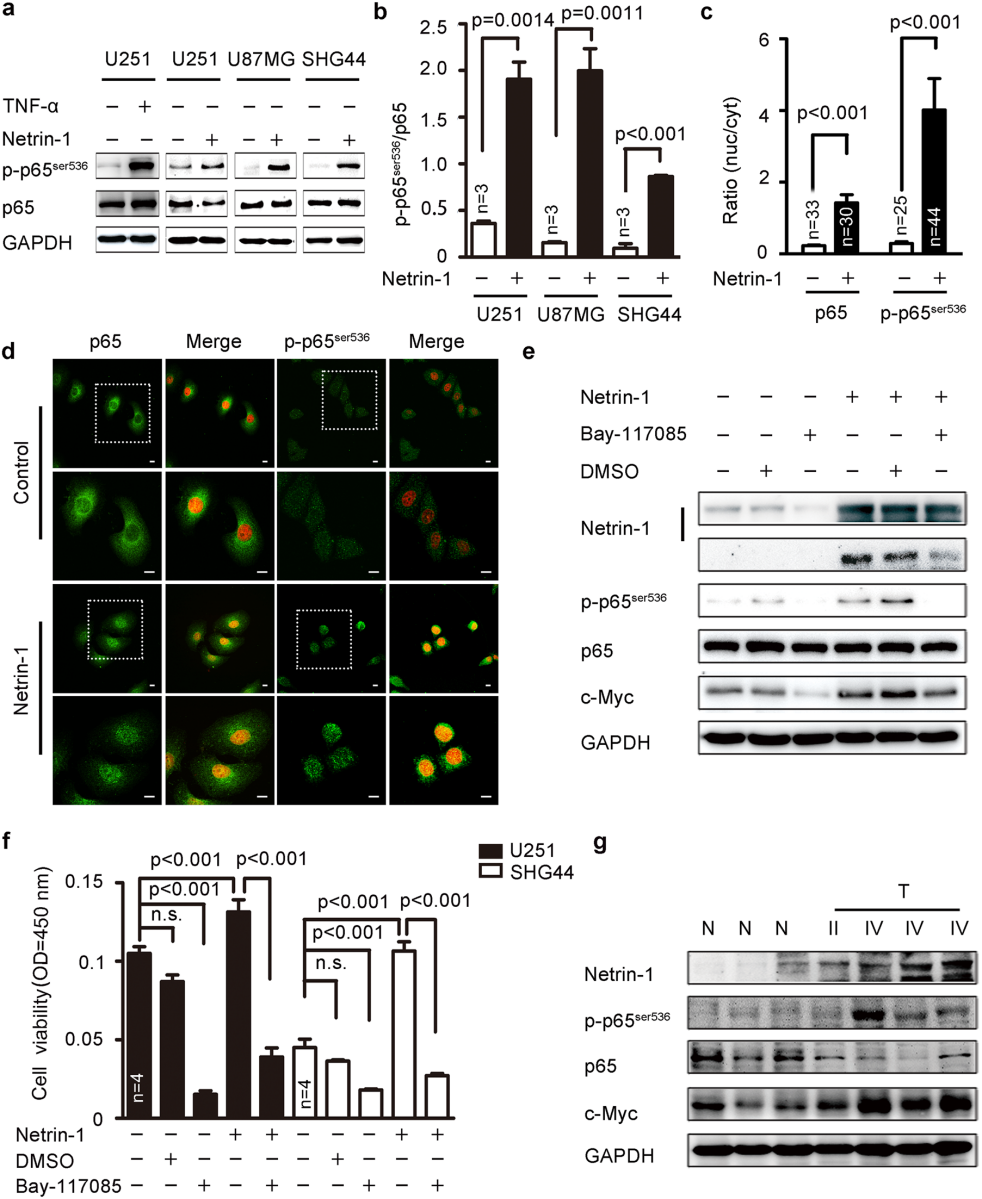
Netrin-1 could activate of the NF-κB signaling pathway. (**a**) Western blotting analyses of p65^ser536^ phosphorylation in U87MG, U251 and SHG44 cells in response to netrin-1 stimulation. TNFα was used as a positive control. (**b**) Quantification of p65^ser536^ phosphorylation using Gel-Pro Analyzer 4 software. Phosphorylation of p65^ser536^ was normalized to total of p65 expression. (**c**) Quantification of cytosolic and nuclear p65 and phosphorylation of p65^ser536^ immunofluorescence results using Image J. (**d**) Subcellular localization of p65 and p65^ser536^ phosphorylation in U251 cells in the absence or presence of netrin-1. DAPI staining is red. Scale bar: 10 μm. Boxed areas in the upper panels were magnified and shown in the corresponding lower panels. (**e**) U251 cells were pretreated with the NF-κB-specific inhibitor Bay117085 (15 µm/L) for 3 h prior to treatment with recombinant human netrin-1 for 30 min. Cells were pretreated with DMSO as a negative control. Netrin-1, p65^ser536^ and c-Myc were detected by western blotting. GAPDH was used as a loading control. (**f**) U251 and SHG44 cells were treated with the NF-κB-specific inhibitor Bay117085 (15 µm/L) for 3 h prior to treatment with recombinant human netrin-1 for 48 h. Cell viability was determined by CCK-8 assay. (**g**) Western blotting analyses of netrin-1, p65^ser536^, total p65 and c-Myc in glioma tumor tissues (T) and normal brain tissues (N) from clinical samples. The data are shown as the mean ± SEM. n.s., P > 0.05.

The *c-Myc* gene is a downstream target of NF-κB. Previous studies have shown that NF-κB regulates c-Myc expression levels via phosphorylation of Ser^536^ in the p65 subunit of NF-κB^37, 38^. Therefore, we examined whether netrin-1 regulated c-Myc through the NF-κB pathway. NF-κB family members are composed of five distinct subunits, including RelA/p65, RelB, c-Rel, p50/p105, and p52/p100. The canonical form of NF-κB is a heterodimer with one p50 subunit and one p65 subunit. Phosphorylation of Ser^536^ in the p65 subunit is known to be a marker of NF-κB signaling pathway activation^39, 40^. We firstly tested whether NF-κB p65^ser536^ was phosphorylated upon netrin-1 stimulation in U251 cells, while TNFα, a well-documented p65 activation inducer, was used as a positive control in the test. We found both TNFα and netrin-1 significantly increased NF-κB p65^ser536^ phosphorylation in U251 cells (Fig. 6a). Similar results were found in U87MG cells and SHG44 cells (Fig. 6a,b). Because NF-kB serves as a transcription regulator, the nuclear translocation of phosphorylated p65 is a key step in its activation^41^. We then studied the nuclear translocation of p65 after netrin-1 treatment using immunofluorescence analysis. Results showed that netrin-1 stimulation dramatically increased the signal intensity of p65 and phosphorylated p65 in the nuclear region (Fig. 6c,d), indicating a functional activation of this protein. In order to determine whether netrin-1-mediated c-Myc activation was dependent on NF-κB, we used an NF-κB-specific inhibitor, Bay117085^42^. The incubation of Bay117085 in cultured cells completely blocked NF-κB p65^ser536^ phosphorylation with or without netrin-1 stimulation. As a result, c-Myc expression was significantly reduced (Fig. 6e). It was worth noting that Bay117085 also inhibited endogenous netrin-1 expression, as previously reported^42^. We then silenced p65 in U251 cells and overexpressed p65 in cultured cells to verify if p65 regulated c-Myc expression at the transcriptional level. Results showed a clear positive correlation between expression levels of p65 and c-Myc (Supplementary Fig. S6). We next examined the effect of Bay117085 on netrin-1-induced cell proliferation. As shown in Fig. 6f, Bay117085 not only reversed the growth advantage stimulated by netrin-1, but also significantly reduced the cell proliferation well below the basal level in both U251 and SHG44 cells.

Furthermore, NF-κB p65^ser536^ phosphorylation and high c-Myc expression corresponded with elevated netrin-1 expression in grade IV clinical glioma samples (Fig. 6g). Taken together, these results suggested that netrin-1 enhanced c-Myc expression via the NF-κB pathway to promote cell proliferation.

### Netrin-1 activation of NF-κB via UNC5A receptor

**Figure 7:**
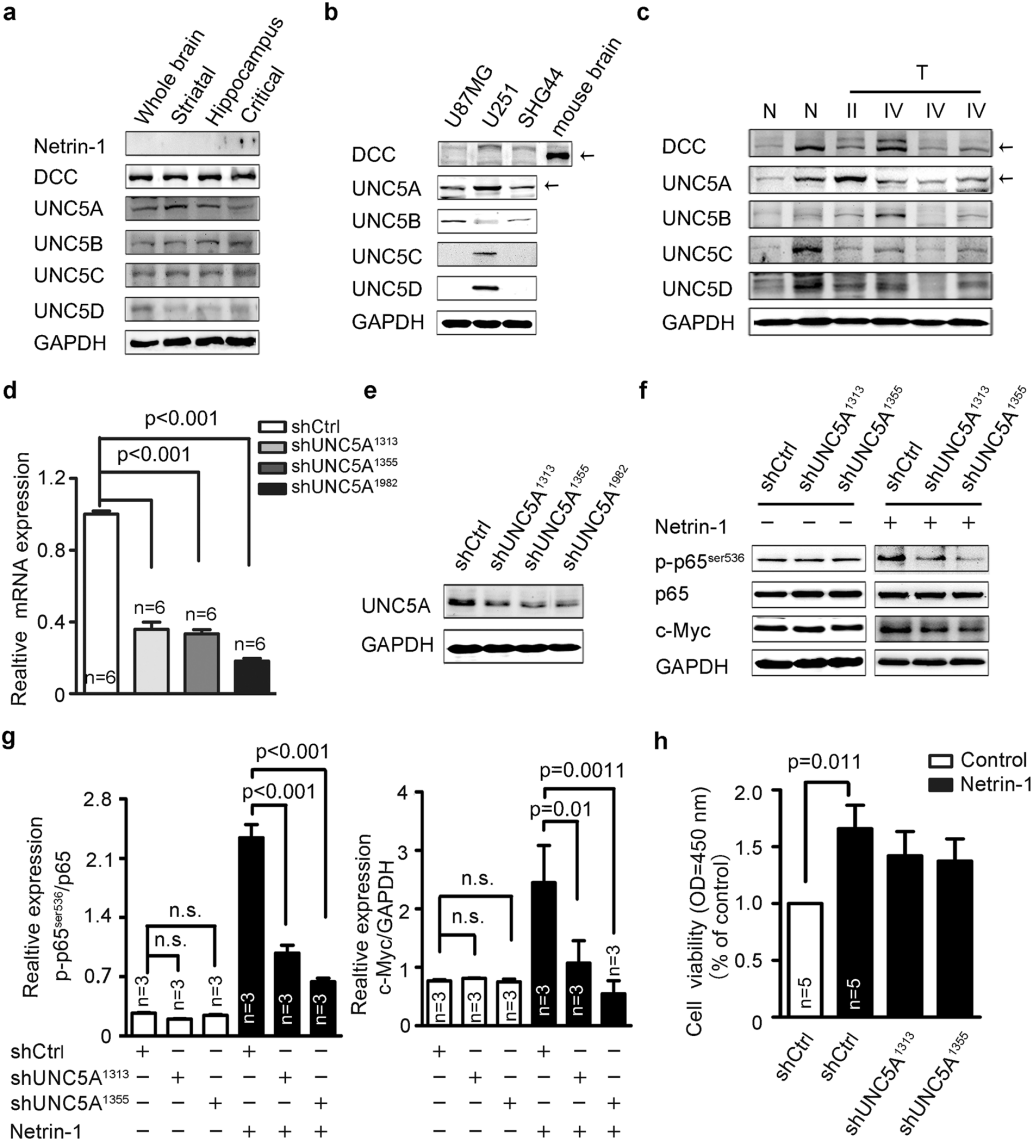
Netrin-1 activation of the NF-κB pathway was dependent on its canonical receptor UNC5A. (**a**) Western blotting analysis of canonical receptors of netrin-1 in the adult mouse brain. (**b**) Western blotting analysis of canonical receptors of netrin-1 in U87MG, U251 and SHG44 cells. (**c**) Western blotting analyses of canonical receptors of netrin-1 in glioma tumor tissues (T) and normal brain tissues (N) from clinical samples. (**d** and **e**) UNC5A mRNA and protein expression levels were determined by real-time PCR and western blotting in U251 cells infected with lentivirus expressing shCtrl or shUNC5A. (**f**) Western blotting analysis of p65^ser536^ and c-Myc in U251 cells infected with lentivirus expressing shCtrl or shUNC5A with or without netrin-1 stimulation. (**g**) Quantification of p65^ser536^ phosphorylation using Gel-Pro Analyzer 4 software. Phosphorylation of NF-κB p65^ser536^ was normalized to total p65, and c-Myc was normalized to GAPDH. (**h**) CCK-8 assays revealed that the addition of netrin-1 promoted cell proliferation. On the contrary, silencing of UNC5A blocked netrin-1-induced cell proliferation. The data are shown as the mean ± SEM.

To determine the mechanism by which netrin-1 promoted glioma proliferation, we examined all the known netrin-1 receptors in the adult mouse brain. As shown in Fig. 7a, the canonical netrin-1 receptors, including DCC and UNC5 homologue family members, were expressed in brains of healthy adult mice while netrin-1 expression was barely detectable. To determine which netrin-1 receptors may play a role in glioma, we determined the expression of these canonical netrin-1 receptors in cultured glioma cells. As shown in Fig. 7b, although DCC was highly expressed in normal mouse brains, none of the three glioma cell lines expressed DCC. While both UNC5A and UNC5B were expressed in all three cell lines, only U251 cells expressed UNC5C and UNC5D. These results were consistent with that relatively higher UNC5A expression was observed in the clinical glioma samples (Fig. 7c). Thus, we hypothesized that UNC5A may be a key receptor involved in netrin-1-mediated tumor growth. We constructed lentiviral-mediated UNC5A shRNAs that effectively knocked down UNC5A expression in U251 cells (Fig. 7d,e). We found silencing of the UNC5A receptor did not alter NF-κB p65^ser536^ phosphorylation or c-Myc expression without netrin-1 stimulation (left panel of Fig. 7f,g), indicating a compensation effect from other netrin-1 receptors. However, UNC5A silencing strongly abolished exogenous netrin-1-induced NF-κB p65^ser536^ phosphorylation and decreased c-Myc expression in the presence of netrin-1 (right panel of Fig. 7f,g). These results were further confirmed in our real-time PCR analyses (Supplementary S7). Netrin-1-induced cell proliferation was partially abolished by silencing of UNC5A (Fig. 7h). These findings suggested that UNC5A should act as a major receptor for netrin-1 to regulate c-Myc expression via NF-κB signaling.

## Discussion

In the present study, we have provided direct evidence netrin-1 in glioma cells increases proliferation and regulates brain tumor growth, implying an important role of netrin-1 in gliomagenesis. We firstly found that netrin-1 was up-regulated in glioma tissues and positively correlated with glioma cell growth. Netrin-1 Knockdown reduced glioma cell proliferation both *in vitro* and *in vivo*. Further mechanistic study revealed that netrin-1 promoted cell proliferation by up-regulating c-Myc expression through activation of the NF-κB signaling pathway using UNC5A as a key receptor.

Previous studies have shown that netrin-1 may act as a survival factor for cancers through inhibiting cell apoptosis, such as breast cancer^22^, colorectal cancer^17^, lung cancer^19^, hepatocyte carcinoma^24^ and neuroblastoma^20^. In contrast, study in pancreatic cancer have reported that netrin-1 inhibited tumor growth by interfering with integrin β4 expression through UNC5B/FAK signaling^43^. Netrin-1 also induced cell apoptosis via up-regulating the tumor suppressor TAp73α in p53-deficient HeLa cells^44^. These studies suggested that netrin-1 may have different functions in different tumor cells. In glioblastoma cells, elevated netrin-1 promoted cell migration and cell invasion^26, 27^. However, little is known regarding how netrin-1 regulates glioma progression. We have demonstrated that netrin-1 was up-regulated in clinical glioma samples. This finding was consistent with metastatic breast cancer and neuroblastoma which also found high expression levels of netrin-1^20, 22^. We also found that up-regulation of netrin-1 was positively associated with glioma grade, malignancy, and progression in clinical glioma cases. A recent study supported our results that netrin-1 expression is associated with poor patient prognosis in grade II-III gliomas^45^. Furthermore, our results demonstrated that up-regulation of netrin-1 was positively correlated with the cellular proliferation index Ki-67, indicating that netrin-1 may play a role in the glioma proliferation. However, this correlation may only apply to primary gliomas because the number of paired recurrent cases in this study was not enough to perform correlation analysis (Supplementary Table S3). In addition, the Ki-67 index usually decreased in the recurrent gliomas due the clinical treatments^46, 47^. Exogenous netrin-1 increased cell proliferation in a dose- and time-dependent manner *in vitro*. This finding was validated in the glioma xenograft mouse models, in which silencing of netrin-1 not only inhibited tumor cells proliferation, but also decreased cellular migration and invasion. In this study, we also examined apoptosis *in vitro* and *in vivo*, but found no significant changes in apoptosis. This observation is consistent with previous studies of netrin-1 knockout mice that have showed complete loss of netrin-1 results in embryonic lethality and severe axon guidance defects without increasing neuronal cell death^48^. Another recent study also discovered that netrin-1 knockdown inhibited glioblastoma stem-like cells proliferation, invasion and angiogenesis^49^. We concluded based on our results and previous studies that netrin-1 is essential for glioma proliferation not only in glioblastoma stem cells, but also in glioma cells, and is also critical in multiple stages of glioma tumorigenesis.

Interestingly, netrin-1 has to interact with its receptors to mediate its effects, such as DCC and UNC5H family, which are dependent receptors. Previous studies found that such receptors were decreased in cancer cells^50–52^. These dependent receptors share the ability to induce apoptosis in the absence of their ligands. Other studies showed that pro-apoptotic activities of these receptors may create a selective cellular survival advantage either to overexpression of netrin-1 or to loss of expression of receptors in colorectal tumorigenesis^17^. Results from aggressive neuroblastoma cells showed that netrin-1 not only conferred a gain in survival, but also led to enhancement of non-apoptotic signaling mediated by netrin-1 receptors^20^. Another recent finding indica﻿ted that netrin-1 could provoke YAP activation through its canonical receptors, UNC5B and DCC^24^, supports this notion. Thus it is tempting to speculate that up-regulation of netrin-1 may enhance survival signaling through its receptors in glioma. The heterogeneity of glioma always resulted in expressing distinctive receptors. We found that UNC5A had a reasonable expression level in the tested glioma cells and glioma patients, suggesting it may mediate the cellular effects of netrin-1 in glioma. This possibility was further supported by the results that knockdown of UNC5A effectively blocked NF-κB activation and inhibited c-Myc expression. Therefore, we have theorized that netrin-1 activates NF-κB via UNC5A to promote glioma cell proliferation. However, we could not exclude the possibility that other receptors may also be involved in this process, as UNC5B/C/D were also expressed at low levels.

It has been shown that there are two main pathways controlling NF-κB activation. One is dependent on IκB kinase-β (IKKβ) and mediated by the p65 transcription factor complex. Another is mediated by the RelB-p52 complex^53^. In our results, we found that netrin-1 induced activation of the NF-kB pathway through enhancing p65^ser536^ phosphorylation and mediating nuclear translocation, suggesting it is an activator of the classical NF-kB pathway in the glioma proliferation. Consistent with previous reports, we also showed that p65 regulated c-Myc expression at the transcriptional level. Thus in our studies we set up a linkage between the elevated netrin-1 expression and the activated NF-κB signaling pathway to promote glioma cells proliferation. However, we could not exclude the possibility that other downstream effectors, such as RelB, may be involved in this process. Recent studies have demonstrated that RelB, an alternative pathway of NF-κB, also plays important roles in glioma invasion^54, 55^. In addition, NF-κB has also been reported to confer a selective advantage for tumor development through up-regulation of netrin-1 expression in colorectal cancer^42^. On the other hand, netrin-1 treatment of human umbilical cord blood-derived mesenchymal led to the activation of NF-κB. These results, combined with our findings, suggested a positive feedback from netrin-1 to NF-κB, which may play an important role in gliomagenesis. However, it requires further investigation to test this hypothesis.

Our results were further supported by a recent publication. While our paper was under review, netrin-1 was reported to associate with poor prognosis in low grade gliomas, which acted as an important regulator for glioblastoma cells in gaining of stemness and enhancing invasive phenotype^45^. We propose based on these results that netrin-1 possibly mediates two key events. Netrin-1 enhances c-Myc expression via NF-κB to promote glioma cell proliferation at least in part via UNC5A. Elevated netrin-1 alters the glioma into more invasive phenotype. Thus, taking into account the multiple roles of netrin-1 in glioma proliferation, migration and invasion, netrin-1 is going to be an innovative anticancer target for glioma treatment.

## Methods

### Cell lines and cell culture

The cell lines used in the present study were obtained from the American Type Culture Collection (Manassas, VA, USA). U87MG, U251, and SHG44 glioma cells, as well as HEK293T cells, were cultured in Dulbecco’s modified Eagle’s medium (DMEM, D5796, Sigma-Aldrich, St. Louis, MO) supplemented with 10% fetal bovine serum (FBS) and 1% penicillin and streptomycin in a humidified 5% CO_2_ incubator at 37 °C. More details can be found in the Supplementary Methods.

### Clinical specimens

Fresh tumor tissues (n = 16, No. 1–16, Supplementary Table S1), adjacent nonmalignant tissues, and normal brain tissues were obtained from the patients who underwent surgery at the First Clinical Hospital of Jilin University (Changchun, China). The use of all human tissue was approved by The Regional Ethical Review Board of Jilin University and Northeast Normal University, Changchun, China and written informed consent were obtained from all patients while they were hospitalized. All experiments were performed in accordance with approved guidelines. Briefly, fresh tumor samples were frozen at −80 °C immediately after surgery and stored for scientific research only. The formalin-fixed paraffin-embedded primary glioma samples (n = 62, No. 17–78, Supplementary Table S1) were diagnosed with glioma based on histology at the First Clinical Hospital of Jilin University.

### Reagents and antibodies

More details can be found in the Supplementary Methods.

### Western blotting

Western blotting was carried out according to the Bio-Rad general protocol for western blotting (BIO-RAD Bulletin 6376 Rev A). For more details, see the Supplementary Methods.

### Real-time PCR

The protocol and primers used are described in the Supplementary Methods.

### Immunocytochemical and immunohistochemical staining

Immunohistochemical (IHC) and immunocytochemical (ICC) staining performed on paraffin-embedded tissues. Adherent cells were used for ICC staining only. Details are included in the Supplementary Methods.

### Cell proliferation assay

Cell growth was assessed using MTT and CCK-8 assays as described in the Supplementary Methods.

### BrdU assay

BrdU incorporation was analyzed according to the Immunofluorescence Protocol for Labeling with BrdU antibody from the Cell Signaling Technology (Danvers, MA, USA). Details are provided in the Supplementary Methods.

### Soft agar colony formation assay

Low melting point (LMP) agarose was used for colony formation. For more details, see the Supplementary Methods.

### Flow cytometry and TUNEL staining

Flow cytometry and TdT-mediated dUTP nick-end labeling (TUNEL) staining are described in the Supplementary Methods.

### Human recombinant netrin-1

The Netrin-1-V5-His construct expressing full-length netrin-1 was a kind gift provided by Dr. Michael Klagsbrun^26^. The construct was transfected into HEK293T cells using polyetherimide (PEI). The culture medium was replaced six to eight hours after transfection. After 24 hours, the cells were cultured in Opti-MEM media. Conditioned medium containing secreted netrin-1 was collected after 48 hours. Recombinant human netrin-1 was purchased from R&D Systems (Millipore, Cat. No. 6419-N1-025).

### Lentiviral packaging and infection of the target cells

More details can be found in the Supplementary Methods.

### Transwell migration and invasion assays

The migration and invasion assays are described in the Supplementary Methods.

### Xenograft tumor mouse models

Male immuno-deficient BALB/c nude mice were used for xenograft tumor models. All protocols were approved by the Guidelines of the Animal Care and Use Committee of Northeast Normal University. All methods and experiments were performed in accordance with relevant guidelines and regulations. Details are described in the Supplementary Methods.

### Statistical analysis

All statistical analyses were performed using SPSS 17.0 software. Student’s *t*-test was used to compare two groups, and one-way ANOVA was used to compare 3 or more groups. The results are presented as the mean ± SEM. A *P* value < 0.05 was considered statistically significant. All graphics were prepared using GraphPad Prism 5 software.

## Electronic supplementary material

Supplementary Information
