# Supplementary material for: Netrin-1 promotes glioma growth by activating NF-κB via UNC5A
Source: Sci Rep. 2017 Jul 14;7:5454. doi: 10.1038/s41598-017-05707-0 (PMC5511130; doi:10.1038/s41598-017-05707-0)
Supplement: Supplementary file 1 — Supplementary Information [file 41598_2017_5707_MOESM1_ESM.pdf]

Netrin-1 promotes glioma growth by activating NF-κB via UNC5A

Jing-Ying Chen[1], Xiao-Xiao He[1], Chi Ma[1], Xin-Min Wu[2], Xin-Lin Wan[1], Zhen-Kai Xing[1], Qing-Qing Pei[1], Xian-Ping Dong[3], Dong-Xu Liu[4], Wen-Cheng Xiong[5,*], and Xiao-Juan Zhu[1,*]

Supplemental data：

Supplementary Methods

Cell culture. Cell lines used in this article were obtained from ATCC (the American Type Culture Collection), where these cell lines were characterized with DNA-fingerprinting and isozyme detection. Cells were immediately expanded and frozen such that they could be revived every 3 to 4 months. Cells were tested every 2 month to ensure negative for mycoplasma contamination with Lonza MycoAlert Mycoplasma Detection Kit (LT07-218).

Reagents. Low melting point (LMP) agarose was purchased from Sangon Biotech (Shanghai, China). Trypsin (1:250) powder (27250-018) was purchased from Gibco. Pentobarbital sodium (P3761) and MTT (M2128, Sigma-Aldrich) were purchased from Sigma-Aldrich. CCK-8 (CK04) was purchased from Dojindo Molecular Technologies. The synthetic NF-κB inhibitor Bay117085 (B5681) was purchased from Sigma-Aldrich. D-Luciferin salt (115144-35-9) were from Gold Biotechnology. TNFα was purchased from PeproTech (300-01A). All other reagents used were purchased from Dingguo Changsheng Biotechnology (Beijing, China).

Antibodies. The netrin-1 antibody was purchased from ENZO Life Science (ALX-210-943-C100, 1:1000 for western blotting). The goat DCC (sc-6535, 1:200), goat UNC5C (sc-54442, 1:300), rabbit

cyclin E (sc-25303, 1:500), rabbit p27 (sc-528, 1:500) and rabbit cdk2 (sc-6248, 1:500) antibodies were obtained from Santa Cruz Biotechnology (Santa Cruz, CA, USA). The UNC5A (SAB2102651, 1:1000) antibody was purchased from Sigma-Aldrich and the UNC5B (ABC89, 1:200) antibody was purchased from Millipore. The rabbit phospho-p65 (Ser536) (93H1, 1:1000), mouse p65 (L8F6, 1:1000), rabbit c-Myc (D84C12, 1:1000 for western blotting, 1:800 for IHC), and rabbit cyclin D1 (#2922, 1:1000) were all purchased from Cell Signaling Technology. The UNC5D (ab58141, 1:250) antibody and the rat BrdU antibody were purchased from Abcam. The rabbit anti-Ki-67 (SA5-14520, 1:500) antibody was purchased from Thermo Fisher Scientific. The mouse anti-GAPDH antibody was purchased from TransGen Biotech.

Western Blotting. Cells and fresh tumor tissues were washed twice with PBS and lysed with RIPA lysis buffer (50 mM Tris-HCl, pH=7.4, 1% sodium deoxycholate, 150 mM NaCl, 1% Triton X-100 and 0.1% SDS) along with c and 1 mM PMSF for 30 min on ice. Then, the lysates were spun down by centrifugation at 12,000 g for 20 min at 4℃. The protein concentrations of the supernatants were determined by Coomassie brilliant blue staining; 5×Laemmli buffer was added to the samples, and then they were boiled for 5 min. The protein lysates were separated by SDS-PAGE and then transferred to PVDF membranes. Hydrophobic PVDF Transfer Membranes (0.45-μm pore size) were purchased from Merck Millipore (Millipore, Cat. No. IPVH00010). The membranes were blocked in 5% BSA at room temperature for 1 h. The primary antibodies were diluted with 2% BSA in TBST and incubated at 4℃ overnight. After incubation, the membranes were washed in TBST for 30 min. Then, the membranes were incubated with HRP-conjugated secondary antibodies (Sigma). Finally, the proteins were detected with Tanon High-sig ECL western Blotting Substrate (Tanon, Shanghai, China, Cat. No. 180-5001). The charge-coupled device (CCD) system used to capture the chemiluminescent signals was the Tanon 5500

high definition low illumination CCD system. The gray scale images were analyzed with Gel-Pro Analyzer 4.0 software.

Real-time PCR. Total RNA was extracted using TriPure isolation reagents (Cat. No. 11667165001, Roche). Complementary DNA was synthesized using transcript One-step gDNA removal and cDNA Synthesis SuperMix (AT-311-03, TransGen Biotech, Beijing, China) according to the manufacturer's protocol. Next, real-time PCR was performed using SYBR Premix Ex Taq^{TM} II (Cat No. RR820A, Takara, Dalian, China) with an ABI StepOnePlus Real-Time PCR System. The following primers were used:

Netrin-1 NM_004822.2	Forward	5'-GAGCCTGAAGACTGCGATTC-3'
	Reverse	5'-ACCGTGAACTTCCACCAGTC-3'
GAPDH NM_002046	Forward	5'-ATGACCCCTTCATTGACCTCA-3'
	Reverse	5'-GAGATGATGACCCTTTTGGCT-3'
UNC5A NM_133369.2	Forward	5'-CATCACCAAGGACACAAGGTTTGC-3'
	Reverse	5'-GGCTGGAAATTATCTTCTGCCGAA-3'
c-Myc NM_002467.4	Forward	5'-CTCCTGGCAAAAGGTCAGAG-3'
	Reverse	5'-TCGGTTGTTGCTGATCTGTC-3'

Immunocytochemical (ICC) and immunohistochemical (IHC) staining. The paraffin-embedded tissue sections used for ICC were subjected to the removal of paraffin with xylene, followed by rehydration with ethanol. For antigen retrieval, the sections were placed in citrate buffer (pH=6.0) at a temperature of 120 ℃ and a pressure of 0.12 MPa for 2 min. A similar procedure was followed for adherent cells, except that neither removal of paraffin nor antigen retrieval was required. Briefly, the sections or the adherent cells were rinsed three times in PBS, blocked with 2% BAS in PBS at room temperature for 2 h, incubated with the primary antibody at 4 ℃ overnight and then incubated with the

appropriate fluorescent secondary antibodies for 1 h at room temperature. The nuclei were stained with 1 mg/mL DAPI (1:200) for 6 min.

For the paraffin-embedded tissues used for IHC, a procedure similar to that performed for ICC was carried out. After antigen retrieval, the sections were washed with PBS three times, using 3% H_2O_2 for 10 min to block endogenous peroxidase activity. After blocking, the sections were incubated with the primary antibody, followed by incubation with the secondary antibody according to the UltraSensitive™ SP (Mouse/Rabbit) IHC kit (KIT-9710, MAB, Fuzhou, China). A peroxidase-based DBA kit (DAB-0031, MAB, Fuzhou, China) was used for detection of the primary antibody. Nuclei were counterstained with hematoxylin. Images were captured using an Olympus FV1000 Viewer laser scanning confocal microscope (Tokyo, Japan) equipped with a 60×1.4 N. A. objective lens.

Cell proliferation assay. Cell growth rates were assessed by the MTT and CCK-8 assays. For the MTT assay, 2×10^3 U87MG cells, 2×10^3 SHG44 cells, and 3×10^3 U251 cells were seeded onto 96-well plates in triplicate. After incubation for the indicated length of time, 20 μl of MTT (5 mg/ml) was added to each well, and the cells were incubated for 4 h at 37 ℃ before removal of the culture medium. The supernatant was removed, and then 100 μl of DMSO was added, and plates were placed on a shaker for 15 min in the dark at room temperature. Cell viability was determined by measuring the absorbance at 490 nm. For the CCK-8 assay, cells were seeded onto 96-well plates. After incubation for the indicated length of time, 10 μl of CCK-8 solution was added to each well, and the cells were incubated for 2 h at 37 ℃. Then, cell viability was determined by measuring the absorbance at 450 nm.

BrdU assay. In brief, 6 h after the addition of BrdU (59-14-3, Sigma-Aldrich, St. Louis, MO), the media were removed, and the cells were fixed with 4% PFA for 10 min. Then, the cells were subjected to three 5 min washes in PBS. Next, 2 M HCl was added to the cells, and the cells were incubated for 30

min at room temperature. The HCl was removed by aspiration. Then, 0.1 M boric acid was added, and the cells were incubated for 2 min. Next, the cells were subjected to three 5-min washes. Then, BSA was added, and the cells were incubated for 2 h at room temperature. After that, the BrdU antibody was added, and the cells were incubated overnight at 4 ℃. After incubation, the cells were washed with PBST 6 times for 5 min each, followed by incubation with the appropriate fluorescent secondary antibodies for 1 h at room temperature. The nuclei were stained with 1 mg/mL DAPI (1:200) for 6 min.

Soft agar colony formation assay. The 0.6% low melting point (LMP) agarose was prepared in deionized H_2O, heated, and cooled. Equal amounts of agarose and DMEM containing 20% FBS were combined. A total of 2 ml of this solution was added to the wells of the 6-well plates, and the plates were incubated for 30 min at 4 ℃ to allow the agar to solidify. This formed the bottom layer of the soft agar. The cells were trypsinized and resuspended at concentrations of 3×10^3 cells per well in DMEM containing 0.3% agarose and 10% FBS. The plates were incubated for 30 min at 4 ℃ to allow the agar to solidify. Once the agar was solid at room temperature, the plates were incubated at 37 ℃ for 16 days. Colonies were counted using a dissecting microscope, and counts were calculated based on three replicate wells for each condition.

Flow Cytometry. Cells were collected by centrifugation, and the supernatant was aspirated. The cells were stained with 50 μg/ml propidium iodide and Annexin V-fluorescein isothiocyanate (Kaiji) following the manufacturer's instructions. The data were analyzed using a flow cytometer.

Lentiviral packaging and infection of the target cells. The scrambled shRNA oligo TTCTCCGAACGTGTCACGT, which has no specific targets in humans, was used as a negative control. The oligos for Netrin-1-shRNA^{1133}, Netrin-1-shRNA^{1153} and Netrin-1-shRNA^{1202} were inserted into the pMT33 lentiviral vector, which harbors a luciferase expression cassette (Shang Sunbio Medical

Biotechnology Co Ltd., Shanghai). The oligos for Netrin-1-shRNA^{619}, Netrin-1-shRNA^{1240}, UNC5A-shRNA^{1313}, UNC5A-shRNA^{1355}, UNC5A-shRNA^{1982}, c-Myc shRNA^{117} and c-Myc shRNA^{118} were inserted into the pLL3.7 lentiviral vector, which has an EGFP expression cassette (Shanghai CPG Biotech Co. Ltd., Shanghai). For the tumor xenograft mouse models, we established a stably transfected U251 cell line that expresses luciferase at a high level for monitoring tumor growth *in vivo*. This is because luciferase expression from the pMT33 shRNA vector was too weak.

The shRNA target sequences are listed below.

Netrin-1 shRNA ^{1133}	5’-GCCACTGCCATTACTGCAA-3’
Netrin-1 shRNA ^{1153}	5’-GGAAGTTCACGGTGAACAT-3’
Netrin-1 shRNA ^{1202}	5’-GCAAGAAGTTCGAAGTGAC-3’
Netrin-1 shRNA ^{619}	5’-GGGTGCCCTTCCAGTTCTA-3’
Netrin-1 shRNA ^{1240}	5’-GCCACTGCCATTACTGCAA-3’
UNC5A shRNA ^{1355}	5’-GCTCCCTGTGGAAGAGTAA-3’
UNC5A shRNA ^{1982}	5’-GCCGGCTGATGATCCCTAATA-3’
c-Myc shRNA^{117}	5’-CACCATCAAGATCAATGGC-3’
c-Myc shRNA^{118}	5’-GACCATCAAGATCAATGGCT-3’

For lentivirus packaging, the target shRNA or scrambled shRNA and the packaging plasmids psPAX2 (Addgene, Cambridge, MA, USA) and pMD2.G (Addgene, Cambridge, MA, USA) were co-transfected into the packaging cell line 293T. Viral supernatants were collected 48 h and 72 h later, and then the supernatants were centrifuged to remove cell debris. Next, the supernatant was filtered using 0.45 μm filters (Millipore) and concentrated using Amicon Ultra centrifugal filters (Millipore 100KD MWCO).

The concentrated virus was used to infect U251, SHG44 and U87MG cells.

Transwell migration and invasion assays. In vitro cell migration and invasion assays were performed as previously described using Transwell chambers (24-well inserts, Corning 8-μm pore size; Corning Costar, Corning, NY, USA). For the migration assay, 2×10^4 cells in 300 μl of DMEM were added to the top chambers, while 700 μl of complete medium was added to the lower chambers. The cells were then incubated for 20 h in a humidified cell culture incubator. After incubation, the cells were washed with PBS three times, fixed with 4% PFA and then stained with 0.1% Crystal Violet. The cells that had not migrated were removed from the upper face of the filters using cotton swabs. Images of five random fields were captured from each membrane, and the number of migratory cells were counted. Similar inserts coated with Matrigel (BD Biosciences) were used to determine invasive potential, and $5x10^4$ cells were used in the invasion assay.

TUNEL staining. An *in situ* cell death detection kit (TMR red, Roche, Cat. No. 11684795910) was used to perform TUNEL staining to evaluate apoptosis. Briefly, paraffin-embedded tissue sections were subjected to the removal of paraffin with xylene and rehydration with ethanol. Then, the tissues were washed with PBS 3 times, followed by incubation with proteinase K (20 μg/ml in 10 mM Tris/HCL) for 10 min at room temperature. Next, tissues were incubated with permeabilization solution (0.1% Triton X-100 in 0.1% sodium citrate) for 2 min on ice. Finally, the tissues were incubated in a TUNEL reaction mixture for 1 h at 37 ℃ in the dark. The nuclei were stained with 1 mg/mL DAPI (1:200) for 6 min.

Xenograft tumor mouse models. Male immuno-deficient BALB/c nude mice weighing 18 - 20 g were purchased from the Wei Tong Li Hua Company in Beijing. The mice were handled in accordance with the Guidelines of the Animal Care and Use Committee of Northeast Normal University. Mice were housed in individually ventilated cages. Cages (including filter tops), bedding and water were autoclaved

before use. The mice were anesthetized and placed in a stereotactic frame with a mouse adaptor. Luciferase-expressing U251 cells (4×10^5) infected with a virus coding for the scramble shRNA or Netrin-1 shRNAs were injected into the right striatum of the brain in five randomly selected mice per group. The injection site for the mice was determined as follows using the position of the bregma: 1 mm anterior, 2 mm lateral and 3 mm intraparenchymal (from the dura). The tumor cells were slowly injected over 7 min. All procedures were carried out under sterile conditions. After injection, all mice were monitored daily. In a few cases, the mice died a few hours after transplantation, and these mice were excluded from the analysis. In the end, the total number of mice in each group was at least four.

The tumor-bearing mice were imaged *in vivo* with bioluminescence on the 16th day after injection and sacrificed on the 27th day when the mice exhibited difficulty eating, cachexia, or action disorder. The brains of the mice were fixed with 4% poly-formaldehyde and processed for paraffin embedding. Then, 5-µm tissue sections were stained by ICC or IHC.

Supplementary Figure

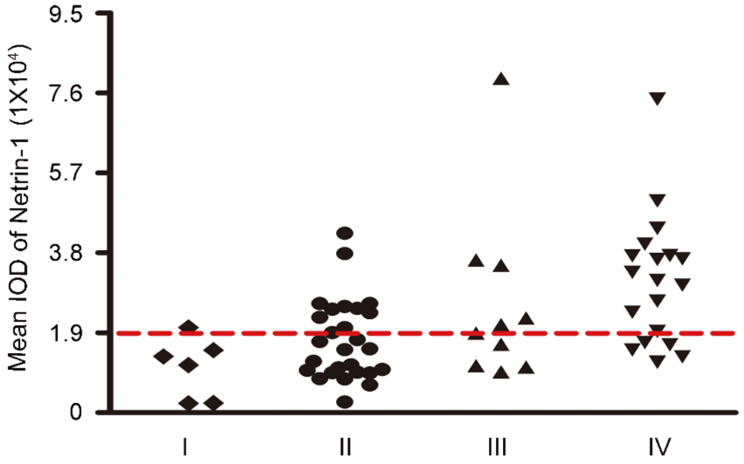

Supplementary Figure S1. The netrin-1 expression in clinical glioma samples. Based on the clinical pathological information, we collected 62 formalin-fixed paraffin-embedded glioma specimens, including grade I (n = 6), grade II (n = 27), grade III (n = 10) and grade IV (n = 19) specimens. The red line represents the median of mean IOD of netrin-1, which was used as a threshold; 16.6% of grade I, 40.7% of grade II, 50% of grade III and 73.7% of grade IV specimens had a mean IOD of netrin-1 higher than the threshold value.

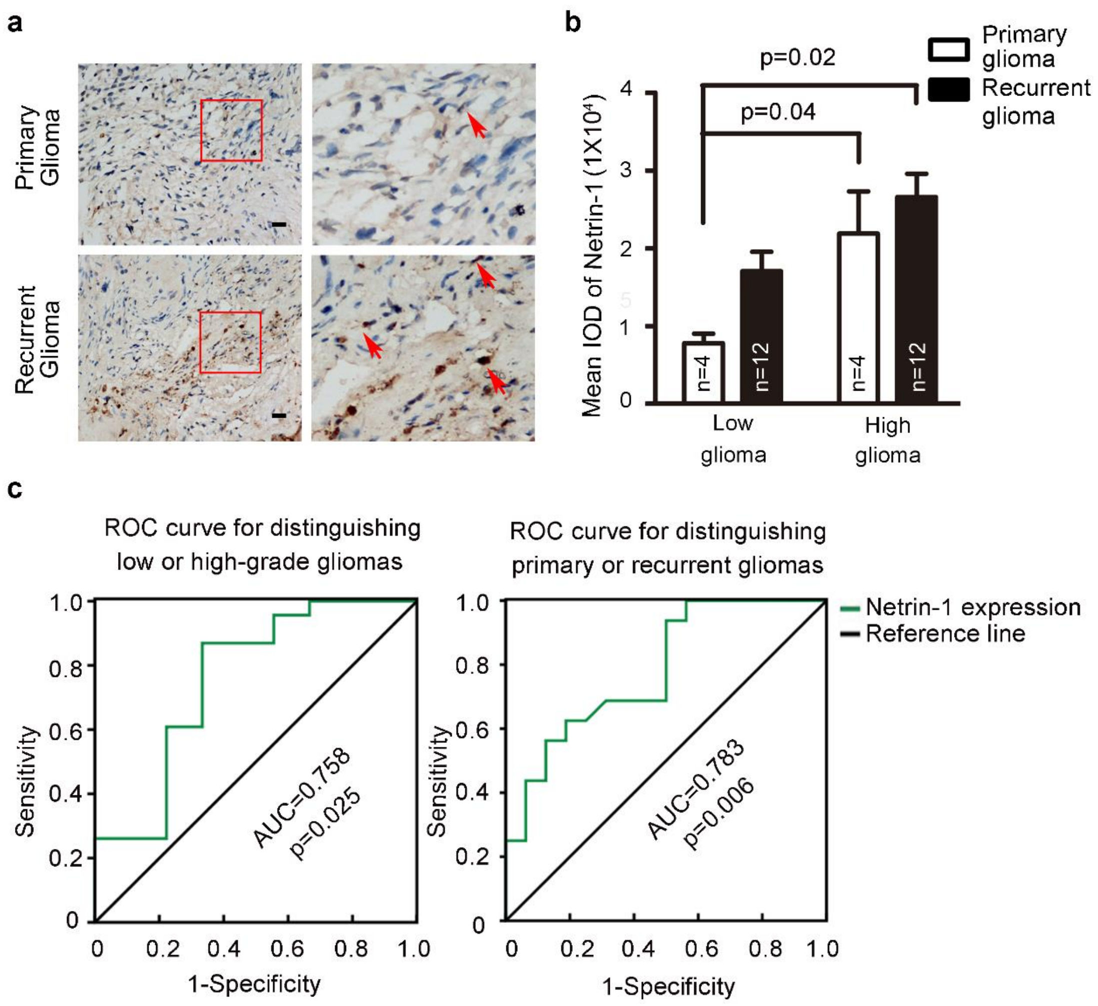

Supplementary Figure S2. Netrin-1 expression in recurrent glioma samples. (a) Representative images of netrin-1 staining (red arrowheads) by IHC in paired primary and recurrent glioma cancer sections. Boxed areas in the left panels were magnified and shown in the corresponding right panels. Scale bar: 50 μm. (b) Relative netrin-1 expression in paired primary and recurrent glioma samples. The mean integrated optical density (IOD) of netrin-1 staining was determined using Image-Pro Plus. (c) ROC analysis showed that netrin-1 expression could discriminate low-grade gliomas from high-grade gliomas (left panel, AUC = 0.758, p = 0.025) and distinguish the primary and recurrent glioma (right panel, AUC = 0.783, p = 0.006). The data are shown as the mean ± SEM.

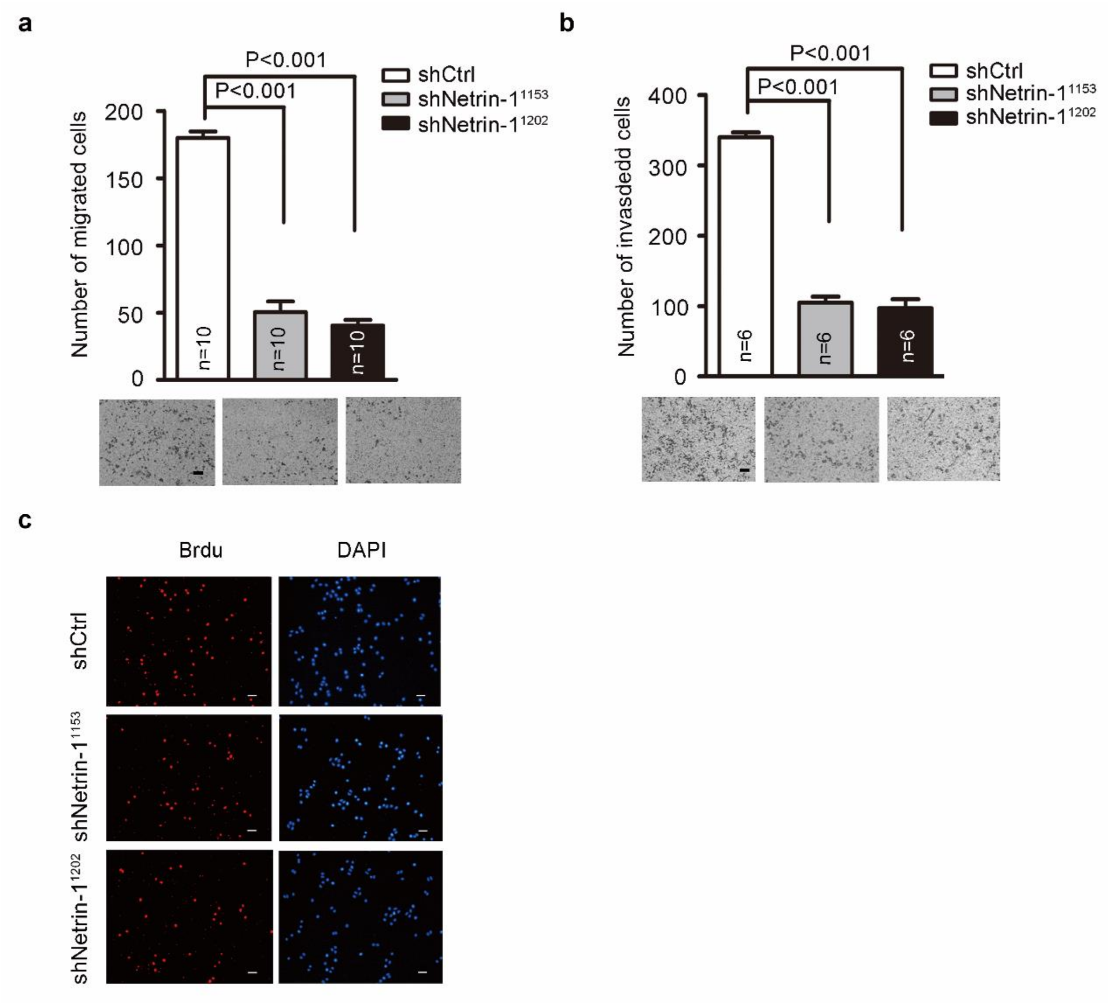

Supplementary Figure S3. **Knockdown of netrin-1 inhibited cell migration, invasion and proliferation.** (a) Cell migration assay in U251 cells infected with lentivirus expressing shCtrl or shNetrin-1. Error bars represent the mean ± SEM of triplicate experiments. (b) Cell invasion assays in U251 cells infected with lentivirus expressing shCtrl or shNetrin-1. Error bars represent the mean ± SEM of triplicate experiments. (c) Representative images showing incorporated BrdU in U251 cells infected with lentivirus expressing shCtrl or shNetrin-1. Scale bar: 50 μm. The data are shown as the mean ± SEM.

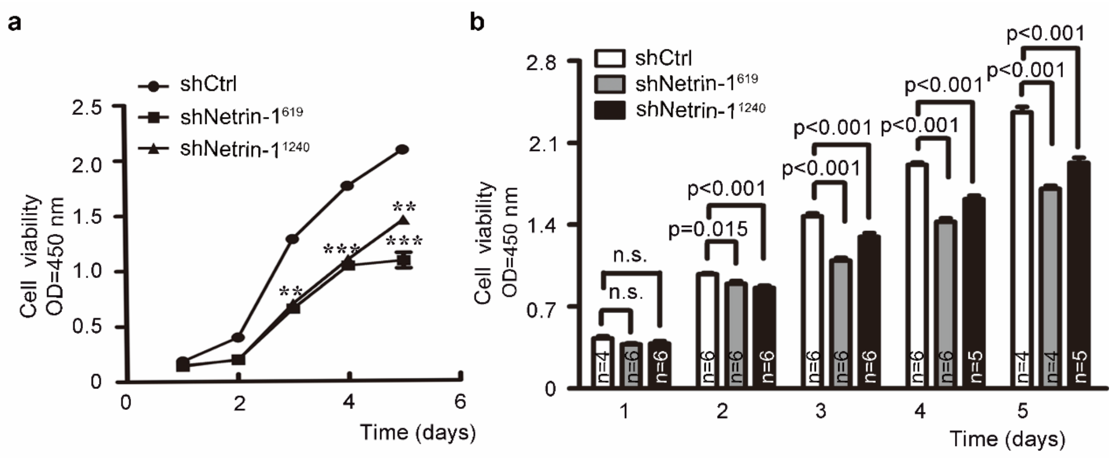

Supplementary Figure S4. Knockdown of netrin-1 inhibited cell proliferation. Cell vitality was determined by CCK8 assay in U251 (a) and U87MG cells (b) infected with lentivirus expressing Netrin-1 shRNAs (shNetrin-1^{619} and Netrin-1^{1240}) or control shRNA. The data are shown as the mean ± SEM. n.s., P>0.5.

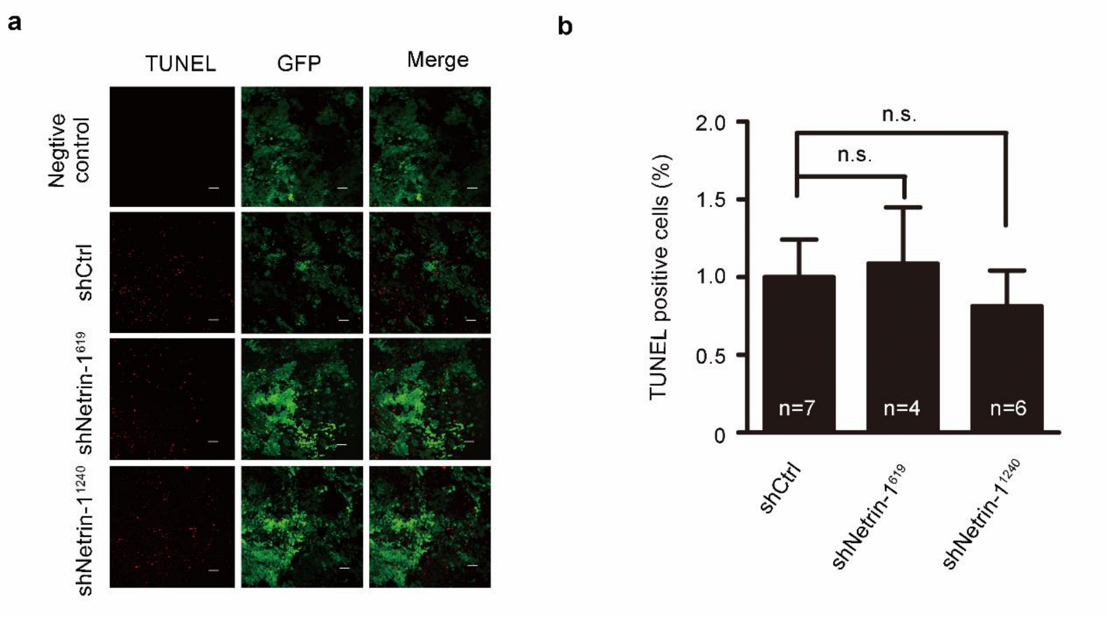

Supplementary Figure S5. Knockdown of netrin-1 couldn't induce cell apoptosis *in vitro*. (a) Representative images of apoptotic cells with TUNEL staining in brain tumors initiated by U251 cells. Scale bar: 50 μm. (b) Quantification of apoptotic cells visualized by TUNEL staining in the three groups. Error bars represent the mean ± SEM; n.s., P>0.5.

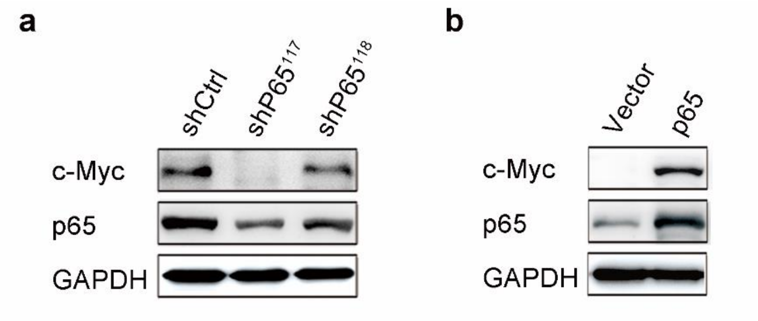

Supplementary Figure S6. P65 regulated c-Myc expression. (a) C-Myc expression was detected by western blotting in U251 cells infected with lentivirus expressing shCtrl or shP65. GAPDH was used as a loading control. (b) Western blotting detected p65 and c-Myc expression after overexpressed p65 in cultured cells. GAPDH was used as a loading control.

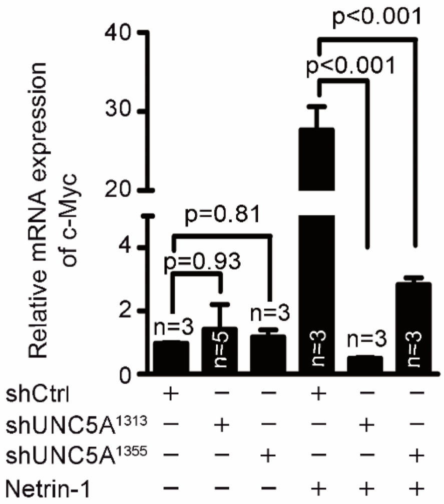

Supplementary Figure S7. Knockdown of UNC5A couldn’t influence c-Myc expression, but could decrease netrin-1-induced c-Myc expression. C-Myc expression was detected by realtime-PCR in U251 cells infected with lentivirus expressing shCtrl or shUNC5A in the absence or presence of netrin-1. Error bars represent the mean ± SEM.

Supplementary Table S1

Supplementary Table S1. The clinical pathologic information of the clinical primary glioma samples.

NO	Age (year)	Sex	Latency	Size(cm)	Grade	Ki-67 (%)	MGMT (%)	GFAP	Mean IOD of Netrin-1	Greater or less than the median
1	20	M	15d	0.5×0.3×0.2	II	2	<20	+	*	*
2	48	M	3m	5.5×2.8×1.7	III	20	<20	+	*	*
3	47	F	*	5×2.5×0.6	II	5	*	+	*	*
4	58	M	10d	5.9×4.8×3.9	III	>10	>20	+	*	*
5	68	M	2m	3.5×2.4×1.6	IV	10	<20	+	*	*
6	31	M	9m	2.9×4.3×3.1	III-IV	5	<20	+	*	*
7	56	F	15d	4×2.5×1.7	IV	40	<20	+	*	*
8	42	M	7d	5×3×1.5	IV	10	<20	+	*	*
9	53	M	5y	4.5×3.5×1	II	4	-	+	*	*
10	57	M	20d	3×2.5×1.2	IV	40	*	+	*	*
11	49	M	1m	4×2×1.5	IV	20	>20	+	*	*
12	44	F	20d	3.7×2.5×1	IV	20	>20	+	*	*
13	28	M	4m	2×1.5×0.7	II	<3	<20	+	*	*
14	53	F	*	2×2.5×1.5	III-IV	40	<20	+	*	*
15	26	M	*	7.5×5.5×4	II	<5	<20	+	*	*
16	31	F	15d	4×3×1.3	II	5	>20	+	*	*
17	18	F	6m	3.2×2.8	I	1	5	+	14948.15	<
18	23	F	20d	2.5×2	I	1	10	+	13410.46	<
19	23	M	1m	3.6×3.6×2.7	I	1	5	+	2323.61	<
20	4	F	2.5y	6.9×5.0×4.8	I	*	*	*	2226.44	<
21	30	M	0.5y	*	I	2	30	+	20272.35	>
22	15	M	5y	5×5.7×4.9	I	*	*	*	11364.77	<
23	43	M	10d	5.1×3.1×3.8	II	5	0	+	24717.37	>
24	38	M	3y	2.5×3.0×3.0	II	1	70	+	9515.46	<
25	61	M	0.5m	3×3×2.5	II	1	80	+	25371.53	>
26	55	F	3y	4.8×4.5×4.4	II	2	0	+	17426.92	<
27	29	M	6m	6×5×4	II	1	1	+	9668.33	<
28	48	F	3y	5×4.6	II	5	70	-	12254.81	<
29	56	M	45d	2.3×2.3×2.5	II	5	30	+	10734.76	<
30	40	M	5y	6×3.3×5	II	1	0	+	9572.39	<
31	16	M	6m	5.3×4.2×4.9	II	1	20	+	15241.17	<
32	25	F	2y	3.5×2.5×1	II	1	40	+	19137.25	>
33	56	M	2y	4×3×4	II	2	10	+	10298.11	<
34	21	M	4m	7.7×7.0×6.2	II	10	20	+	24873.73	>
35	39	M	1m	5.5×5.1×2.7	II	*	*	*	23834.03	>
36	33	F	6m	3.4×4.2	II	1	20	+	16991.15	<
37	36	M	1m	3×3	II	1	20	+	11396.42	<

38	34	M	2m	5.8×4.1	II	2	40	+	15062.62	<
39	33	F	3y	2.5×3.4×2.6	II	2	*	+	6626.08	<
40	30	F	1m	4.5×5.0×6.0	II	10	20	+	26073.22	>
41	57	M	6m	4.4×3.7×3.2	II	5	40	+	20314.23	>
42	48	M	1m	4×3×3	II	1	80	+	2536.91	<
43	26	F	3m	5×4	II	5	20	+	8124.85	<
44	20	M	5y	5.5×5.5×6	II	20	70	+	10114.03	<
45	59	M	2y	2.5×2.0×2.2	II	3	50	+	42805.64	>
46	33	F	10m	1.3×1.7	II	1	5	*	38008.76	>
47	32	M	2m	4.5×5×5	II	5	10	+	8039.22	<
48	8	M	1.5m	3.9×2.9×3.7	II	1	50	*	22737.98	>
49	4	M	1y	6.9×4.5×5.4	II	*	*	+	26083.47	>
50	34	F	7y	7×9×9.4	III	5	0	+	19014.1	<
51	73	F	2m	8×5.7	III	10	10	+	21002.66	>
52	41	M	4m	7×7	III	2	20	+	16283.19	<
53	33	M	3m	2.2×1.8×1.0	III	5	10	-	9742.47	<
54	33	F	1m	2.8×5.3×4.1	III	2	10	+	10879.56	<
55	55	M	6m	3×4×4	III	5	40	+	11224.57	<
56	55	F	1m	4×5×4	III	20	0	+	35196.03	>
57	55	F	7d	4.43×3.34	III	20	50	+	22590.38	>
58	57	F	10y	5×4.5×4.5	III	20	10	+	79809.54	>
59	40	F	1m	4.3×4.6×4.8	III	30	20	+	36422.93	>
60	14	M	15d	6×6×5	IV	30	80	+	26650.14	>
61	51	M	2y	2.8×3.2×3.6	IV	10	80	+	30479.25	>
62	59	F	20d	2.6×3.5×4	IV	20	20	+	50513.36	>
63	37	M	1y	5×3.5×3.2	IV	20	20	+	23960.29	>
64	41	F	20d	5.4×4×5	IV	10	10	+	36757.37	>
65	27	F	3m	4×5×5.5	IV	80	10	+	13383.62	<
66	63	M	10m	3.3×6.6×5.4	IV	5	0	+	37447.51	>
67	25	F	1m	5.1××4.1×4	IV	5	20	+	37547.1	>
68	51	M	1.5m	4.2×2.9×3.8	IV	15	20	+	31548.32	>
69	76	M	1y	5.3×5.6×4.9	IV	*	20	+	36601.44	>
70	20	F	1m	3.6×5.8×3.3	IV	10	30	+	74806.94	>
71	12	M	20d	3 ×2.5 ×2	IV	80	*	+	40254.62	>
72	55	F	2y	3 ×3 ×2	IV	5	80	+	33533.09	>
73	49	F	1m	2.7 ×2.4 ×2.1	IV	10	0	+	16244.59	<
74	53	M	10d	3.2 ×2.2 ×2.6	IV	10	40	+	43971.18	>
75	73	F	10d	1.1 ×1.3	IV	20	10	+	19385.92	>
76	54	M	7d	6 ×5 ×4	IV	10	20	+	14962.53	<
77	45	F	1m	2.6 ×3.8	IV	20	20	+	16730.32	<
78	65	F	7d	9.8 ×4.5	IV	20	80	+	12072.39	<

M, Male; F, Female; *, have no available data; d, day; m, month; y, year.

No.1-16 is the fresh glioma samples. They are used for western blotting, real-time PCR and IHC.

No.17-78 is the formalin-fixed paraffin-embedded glioma specimens. They are only used for IHC.

Median of the mean IOD of netrin-1 is 19075.

Supplementary Table S2

Supplementary Table S2. Netrin-1 expression in normal brain and glioma.

Data were obtained from the cancer microarray database Oncomine (www.oncomine.org).

Normal vs. Carcinoma*	Sample number	Median	Fold change#	p-value	References
Brain<br>Diffuse Astrocytoma	23<br>7	0.104<br>1.737	3.327↑	3.73E-04	(Sun et al., 2006)[45]
Brain<br>Oligodendroglioma	23<br>50	0.104<br>1.904	3.145↑	2.52E-14	
Brain<br>Anaplastic Astrocytoma	23<br>19	0.104<br>1.935	3.735↑	6.07E-14	
Brain<br>Anaplastic Oligoastrocytoma	6<br>4	-0.422<br>1.81	4.545↑	3.67E-06	(French et al., 2006)[46]
Brain<br>Anaplastic Oligodendroglioma	6<br>23	-0.422<br>1.611	3.289↑	8.87E-11	
Brain<br>Glioblastoma	23<br>81	0.104<br>2.01	3.823↑	2.91E-19	(Sun et al., 2006)[45]
Brain<br>Glioblastoma	4<br>80	0.406<br>1.543	2.160↑	8.01E-06	(Murat et al., 2008)[47]
Brain<br>Glioblastoma	4<br>80	0.003<br>-0.127	-1.119↓	1.35E-04	(Murat et al., 2008)[47]
Brain<br>Glioblastoma	23<br>81	-0.422<br>-0.882	-1.469↓	1.00E-03	(Sun et al., 2006)[45]
Brain<br>Anaplastic Oligodendroglioma	6<br>23	0.104<br>1.904	-1.183↓	2.00E-03	(French et al., 2006)[46]
Brain<br>Anaplastic Astrocytoma	23<br>19	-0.426<br>-1.201	-1.807↓	3.00E-03	(Sun et al., 2006)[45]
Brain<br>Oligodendroglioma	23<br>50	-0.426<br>-1.048	-1.631↓	3.35E-04	
Brain<br>Diffuse Astrocytoma	23<br>7	-0.422<br>1.611	-1.52↓	4.70E-02	

* All studies with a p value < 0.05 in Student's *t*-test were shown.

# indicated an increased or decreased expression in glioma tumors compared with the normal brain tissues.

Supplementary Table S3

Supplementary Table S3. The clinical pathologic information of paired primary and recurrent glioma samples.

NO		Age (year)	Sex	Latency	Size(cm)	Grade	Ki-67 (%)	MGMT (%)	Mean IOD of Netrin-1
1	P	51	M	1y	5.6×3.9×4.1	II	*	*	5508.2084
	R	52	M	2m	5.5×4.6	II	*	*	10491.275
2	P	53	M	2m	4.1×5.2×5.1	II	60	0	3748.5848
	R	53	M	10d	2.0×4.1	III	*	*	3544.0833
3	P	39	M	2m	4×5×3	II-III	30	0	17593.084
	R	40	M	9m	3×3	II-III	10	80	14753.04
4	P	54	F	15d	4.5×4.8	II	10	5	6038.3896
	R	54	F	7d	3.5×4×3	III	5	30	11725.668
5	P	55	M	*	4.7×2.5×4	III	1	80	9811.5095
	R	55	M	9m	3×3.5	III	*	*	15071.896
6	P	48	F	1y	3×2.5×1.2	III	5	30	19652.038
	R	50	F	4m	2.5×2.7	IV	*	*	21320.309
7	P	43	M	7d	3.5×2.5	III	80	20	22751.559
	R	43	M	4m	4.5×5	IV	5	3	34976.368
8	P	53	M	1w	5×5.5×6	IV	*	*	22705.605
	R	55	M	1.5y	5.5×6×5.5	III-IV	30	5	7576.7376
9	P	28	M	1w	4.5×3×4	III	5	*	17587.078
	R	30	M	1y	6×6.5×7.5	III-IV	60	30	35975.475
10	P	52	F	7d	5×7	IV	60	20	10023.168
	R	53	F	13m	6.2×6.5	III-IV	*	*	14263.358
11	P	44	M	10d	3.5×5.2	IV	*	*	28539.89
	R	45	M	30d	3.2×4.0	IV	30	10	44900.193
12	P	38	M	2m	4.9×3.3×3.4	II	1	5	10862.208
	R	39	M	1.5y	5.5×4.8×4.1	IV	6	10	30840.54
13	P	48	F	2y	3×4×4	III	10	-	17836.229
	R	50	F	0.5m	3.5×3.1×4.1	IV	40	40	28262.249
14	P	37	M	3m	2.4×2.6×2.9	III-IV	50	0	33416.626
	R	38	M	6m	7.5×4.8×5.7	IV	10	20	16490.026
15	P	55	M	10d	3.5×4.0	IV	20	10	33339.261
	R	56	M	1m	6.0×3.5	IV	20	10	32845.234
16	P	44	M	2y	8.1×7.6	II	*	*	8660.8226
	R	46	M	2y	4×5×3	II	*	0	31485.784

P, primary glioma; R, recurrent glioma; M, Male; F, Female; *, have no available data; d, day; w, week; m, month; y, year.
